# Germline *Jak2*-R1063H mutation interferes with normal hematopoietic development and increases risk of thrombosis and leukemic transformation

**DOI:** 10.1038/s41375-025-02737-w

**Published:** 2025-08-21

**Authors:** Veronika Zimolova, Monika Burocziova, Linda Berkova, Srdjan Grusanovic, Jan Gursky, Lubos Janotka, Petr Kasparek, Alena Pecinova, David Kundrat, Dusan Hrckulak, Jakub Onhajzer, Ivana Jeziskova, Lucie Nekvindova, Barbora Weinbergerova, Sarka Pospisilova, Michael Doubek, Meritxell Alberich-Jorda, Vladimir Korinek, Vladimir Divoky, Lucie Lanikova

**Affiliations:** 1 Laboratory of Cell and Developmental Biology, Institute of Molecular Genetics of the Czech Academy of Sciences, Prague, Czech Republic; 2 Laboratory of Hematooncology, Institute of Molecular Genetics of the Czech Academy of Sciences, Prague, Czech Republic; 3 Department of Biology, Faculty of Medicine and Dentistry, Palacky University, Olomouc, Czech Republic; 4 Czech Centre for Phenogenomics & Laboratory of Transgenic Models of Diseases, Institute of Molecular Genetics of the Czech Academy of Sciences, Prague, Czech Republic; 5 Laboratory of Bioenergetics, Institute of Physiology of the Czech Academy of Sciences, Prague, Czech Republic; 6 Department of Genomics, Institute of Hematology and Blood Transfusion, Prague, Czech Republic; 7 Department of Internal Medicine - Hematology and Oncology, University Hospital Brno and Faculty of Medicine, Masaryk University and ERN EuroBloodNet Centre, Brno, Czech Republic; 8 ANOVA CRO Ltd., Brno, Czech Republic; 9 Center of Molecular Medicine, CEITEC, Masaryk University, Brno, Czech Republic; 10 Department of Medical Genetics and Genomics, University Hospital Brno and Faculty of Medicine, Masaryk University, Brno, Czech Republic; 11 Childhood Leukaemia Investigation Prague, Department of Pediatric Haematology and Oncology, 2nd Faculty of Medicine, University Hospital Motol, Charles University, Prague, Czech Republic

**Keywords:** Myeloproliferative disease, Risk factors

## Abstract

The acquired *JAK2*-V617F mutation plays a causal role in myeloproliferative neoplasms (MPN). Weakly activating *JAK2* germline variants have been associated with MPN risk, but the underlying mechanisms remain unclear. We previously identified the *JAK2*-R1063H germline variant, which contributes to hereditary MPN and increased disease severity in essential thrombocythemia. Here, we studied alterations in hematopoiesis in *Jak2*-R1063H knock-in mice. The *Jak2*-R1063H mouse cohort exhibited increased mortality, stimulated thrombopoiesis and elevated D-dimers levels, indicative of thrombotic complications. Bone marrow analysis revealed myeloid bias, enhanced megakaryopoiesis and activation of inflammatory signaling. Transcriptional and functional assays of hematopoietic stem cells suggested their accelerated aging and functional decline. The Egr1 transcriptional network, including the *Thbs1* gene, progressively increased in aging mice, reinforcing alterations initiated by Jak2/Stat signaling. In murine acute myelogenous leukemia models, the *Jak2*-R1063H cooperated with a driver oncogene in promoting leukemogenesis. Germline *JAK2*-R1063H was found in 10 of 200 MPN patients from local hematology centers, with a higher minor allele frequency compared to healthy controls. Patients harboring *JAK2*-R1063H variant exhibited an increased incidence of thrombotic complications and disease progression with shortened survival. In conclusion, our findings identify the *JAK2*-R1063H germline variant as a risk factor for MPN development, thrombotic complications, and leukemic transformation.

**Figure:**
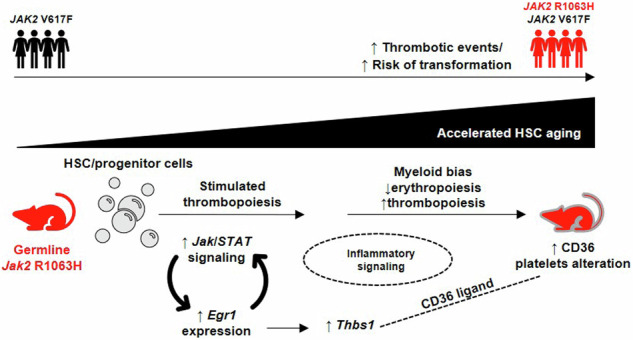
Our study, which involves a mouse model and a cohort of 200 MPN patients, characterizes the *JAK2*-R1063H germline mutation as a risk factor for MPN development, thrombotic complications, and leukemic transformation. These findings may have important clinical implications for managing MPN patients carrying the *JAK2*-R1063H germline variant.

Our study, which involves a mouse model and a cohort of 200 MPN patients, characterizes the *JAK2*-R1063H germline mutation as a risk factor for MPN development, thrombotic complications, and leukemic transformation. These findings may have important clinical implications for managing MPN patients carrying the *JAK2*-R1063H germline variant.

## Introduction

The Janus kinase 2 (*JAK2*) gene encodes for a tyrosine kinase responsible for mediating a critically important signaling in immune and hematopoietic cells [1]. The most frequently occurring gain-of-function *JAK2* mutation, V617F, gives rise to a constitutively active JAK2 kinase, which drives the JAK/STAT signaling that leads to excessive proliferation and survival of myeloid progenitor cells in most of the Philadelphia chromosome-negative classical myeloproliferative neoplasms (MPNs) [2]. Multiple studies have demonstrated that besides the somatic mutation(s), there is a substantial hereditable component, linked to the risk of MPN[3–5], and germline variations in *JAK2* itself, such as the *JAK2* GGCC haplotype, have been associated with *JAK2*-V617F somatic mutation acquisition and the development of MPNs [6, 7]. Also, non-canonical, weakly activating germline *JAK2* variants may predispose progenitor cells to acquire *JAK2*-V617F and accelerate MPN progression to acute myeloid leukemia (AML) [8, 9]. Cases of hereditary thrombocytosis associated with some germline *JAK2* variants indicate a distinct impact of *JAK2* variants compared to somatic *JAK2* mutations [10–12]. The *JAK2-*R1063H germline variant, functionally characterized in cooperation with *JAK2*-E846D, contributed to hereditary MPN with erythrocytosis and megakaryocytic atypia [13]. Mambet et al. [14] showed that patients with essential thrombocythemia (ET) carrying *JAK2-*R1063H and *JAK2*-V617F mutations had increased JAK2 signaling and disease severity, with 28% of them experiencing thrombotic events. The study also demonstrated that while most of the patients with the *JAK2*-R1063H mutation were heterozygous germline carriers, rare patients were (nearly) homozygous for the R1063H mutation and in rare cases the R1063H was an acquired somatic mutation [14]. Additionally, whole exome sequencing identified *JAK2*-R1063H in a patient with venous thromboembolism, suggesting it as a potential disease-causing variant [15]. Evaluation of the risk of MPN-associated thrombosis typically considers quantitative changes in platelets [16], but metabolic alterations mediating aberrant platelet activity and function may also play a crucial role, potentially driving hyperreactivation and increasing thrombotic risk in these patients [17]. However, the mechanism of thrombosis associated with weakly activating *JAK2* germline mutations has not been described, nor it is known whether these variants modulate hematopoietic stem cell (HSC)/progenitor function as proposed for other contributors to germline MPN risk [3]. In this context, no study to date has investigated whether the *JAK2*-R1063H variant may promote a non-hierarchical platelet differentiation pathway, in which platelet-biased HSCs give rise directly to megakaryocyte progenitors (MkPs), bypassing canonical multipotent intermediates [18, 19], and leading to overactive platelets that increase the risk of thrombosis in the elderly [19]. To study the biology of the *JAK2*-R1063H variant, we generated CRISPR/Cas9-edited *Jak2*-R1063H mice and provide a phenotypic and molecular characterization of this model.

## Materials and methods

### Ethics approval and consent to participate

The study was conducted in accordance with the principles of the Declaration of Helsinki. The project was approved by the Ethics Committee of University Hospital Brno. Reference number 22-240620/EK and approval number 102/20 dated 24 June 2020. Written informed consent for participation and publication of results was obtained from all enrolled patients and/or their legal guardians. For in vivo work in mice, studies were carried out in compliance with The Ministry of Agriculture of the Czech Republic and protocols approved by the Czech Academy of Sciences Animal Welfare and Ethical Review Advisory Body (protocol 67/2020).

### Mice

The mouse model bearing *Jak2*-R1063H mutation was created using microinjection of synthesized 5 μM ssODNs (87 bp) containing the R1063H mutation, 5 μM sgRNA mRNA and 100 ng/μl Cas9 protein into C57BL6/N-derived zygotes. In this study, 10–14- and 52–62-week-old mice were referred to as young (3 M) and old (12 M), respectively. In some experiments, 75- to 80-week-old mice were analyzed, referred to as 18 M. Since the quantification of the R1063H allele in the patient DNA samples indicated both heterozygous and homozygous state [14], we bred and analyzed the knock-in *Jak2*-R1063H homozygous mice (depicted as RH/RH; unless otherwise stated, the designation *Jak2*-R1063H mice refers to this genotype) to enhance the potential effect. In some experiments, we also analyzed heterozygous *Jak2*-R1063H mice (depicted as m/RH). Additional methods are described in the Supplemental File.

## Results

### *Jak2*-R1063H mutant mice exhibit stimulated thrombopoiesis and age-related decline of erythropoiesis

**Fig. 1:**
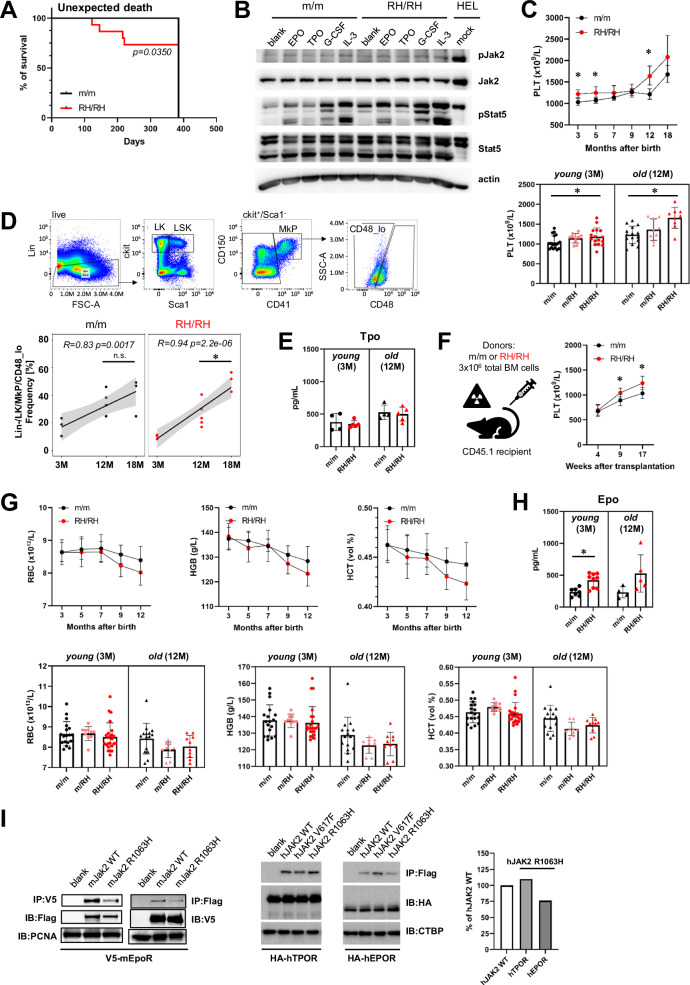
Characterization of the *Jak2*-R1063H mouse model. **A** Increased mortality rate of the mutant mice. Kaplan-Meier survival analysis of m/m (wt) (*n* = 15) and RH/RH (*n* = 15) mice. Mantle-Cox test was used to assess statistical significance of obtained results (*p* = 0.0350). **B** Increased baseline (blank line) and cytokine-stimulated Jak2/Stat5 activation in lysates of total BM cells obtained from RH/RH 3-month-old animals (*n* = 2, pooled cells), when compared to age-matched controls (*n* = 2), identified by WB analysis. **C** PLT counts for m/m (wt) and RH/RH are shown at the indicated times (up); comparison of PLT counts for m/m (wt), heterozygous and homozygous *Jak2*-R1063H mice at 3 and 12 months of age (down), *n* ≥ 10 mice per group, per each time point. **D** Phenotypic MkPs stratification based on CD48 expression. Representative flow cytometry plot (up) and quantification of CD48_low non-hierarchical MkPs at indicated time-points (*n* ≥ 3 mice per group) (down). **E** Tpo concentration in plasma (*n* ≥ 4 mice per group). **F** Workflow of transplantation of unsorted *Jak2*-R1063H-positive or wt BM cells to lethally irradiated C57BL/6NCrl recipients and PLT counts after transplantation (*n* ≥ 10 mice per group). **G** RBC, hemoglobin (HGB) and hematocrit (HCT) levels at the indicated times (*n* ≥ 10 mice per group, per each time point) (up). Comparison of RBC, HGB and HCT counts for m/m (wt), heterozygous and homozygous *Jak2*-R1063H mice at 3 and 12 months of age, *n* ≥ 10 mice per group, per each time point (down). **H** Epo concentration in plasma (*n* ≥ 4 mice per group). **I** Altered coupling of mouse (m) Jak2-R1063H or human (h) JAK2-R1063H to hematopoietic receptors. Mouse Jak2-Flag and human JAK2-Flag mutants were transiently expressed in HEK293 cells in which mouse V5-tagged Epor and human HA-tagged EPOR/TPOR were stably expressed. Interaction was examined by co-immunoprecipitation with Flag/V5 antibody. Immunoblot band intensity was quantified by ImageJ software and normalized to loading control and human JAK2 wt. All data are presented as mean ± SD and unpaired t-test with Welsch’s correction was used for group comparison. *p < 0.05.

The mice were born in normal (expected) frequency without marked changes in spleen size and bone marrow (BM) cellularity (Supplemental Fig. 1A). However, we observed a relatively high frequency of sudden death in the cohort of *Jak2*-R1063H mice with median overall survival (OS) of 215 days (5/15) in comparison with the wt group (0/15) (Fig. 1A). We analyzed functionality of the *Jak2*-R0163H mutation in this in vivo model. Western blot (WB) analysis showed that Jak2 protein in unsorted BM cells was comparable between *Jak2*-R1063H and wt mice, but BM cells stimulated with EPO, TPO, G-CSF and IL-3 resulted in higher activation of Stat5 in the mutant cells (Fig. 1B). We observed phosphorylated Stat5 also in the absence of added growth factors in R1063H BM cells, suggesting weakly activated signaling in ligand-unstimulated cells (Fig. 1B). This experiment showed that the *Jak2*-R0163H mutation has expected regulation in mice with the ability to overstimulate downstream receptor signaling.

The *Jak2*-R1063H mice displayed significantly increased platelet (PLT) counts when compared to wt mice during life-span (Fig. 1C top), but only with age the counts exceeded the physiological levels for C57BL/6N background [20]. Analysis of 3- and 12-month-old heterozygous *Jak2*-R1063H animals (m/RH) showed intermediate phenotype, supporting a dose-dependent effect of the *Jak2*-R1063H mutation and excluding an unexpected phenotype associated with the homozygous state (Fig. 1C bottom). To investigate whether the observed hematopoietic alterations might be driven (at least partially) by an expansion of platelet-biased HSCs (i.e. recently described non-hierarchical megakaryopoiesis pathway) [18, 19] we measured the expansion of Lin^−^/cKit^+^/CD41^+^/CD48^−^ cells. This expansion was pronounced from 12 months of age, preceding the age-associated increase observed in m/m (wt) animals (Fig. 1D**)**. These findings suggest that *Jak2*-R1063H progressively activates a platelet-biased differentiation trajectory through involvement of direct pathway from HSCs to MkPs [18, 19].

The plasma thrombopoietin (Tpo) level was normal (Fig. 1E). To further confirm that the observed trend towards increased PLTs is a cell autonomous defect, unsorted BM cells from wt or *Jak2*-R1063H mice were transplanted into the lethally irradiated recipients. The onset of increased PLTs was observed 9 weeks after transplantation and persisted till 17 weeks, when the experiment was terminated (Fig. 1F, Supplemental Fig. 1B).

The red blood cell (RBC) characteristics were normal but with more pronounced signs of anemia in aged *Jak2*-R1063H group than in wt mice (Fig. 1G top) and analysis of 3- and 12-month-old heterozygous *Jak2*-R1063H animals (m/RH) showed an intermediate phenotype, further supporting a dosage-dependent impact of the mutation also on erythroid parameters (Fig. 1G bottom). We also observed normal maturation of RBCs (data not shown), non-altered iron metabolism (the exception was increased ferritin in old *Jak2*-R1063H mice, Supplemental Fig. 1C), but increased erythropoietin (Epo) levels (Fig. 1H). White blood cell (WBC) parameters remained unaltered at all examined time points (Supplemental Fig. 1D).

Although the WB analysis (Fig. 1B) revealed activation caused by the *Jak2*-R0163H mutation downstream of EpoR and TpoR, it was documented in the presence of excess of ligand, with limited functional relevance for in vivo context. We hypothesized, that the observed phenotype in thrombopoiesis/erythropoiesis could be (at least partially) modulated by different coupling affinity of the Jak2-R0163H kinase to EpoR and TpoR, as seen with human G-CSFR, when JAK2-V617F/R1063H mutant couples to G-CSFR with higher affinity than JAK2-V617F [14]. We performed co-immunoprecipitation experiments in unstimulated HEK293 cells stably expressing mouse EpoR and TpoR. As shown in Fig. 1I left, less EpoR co-immunoprecipitated with Jak2-R1063H. Due to technical issues with very weak TpoR signal, we were unable to reliably evaluate the TpoR/Jak2-R1063H co-IP results (data not shown). However, when we used HEK293 cells stably expressing human EPOR and TPOR, more TPOR and less EPOR co-immunoprecipitated with human JAK2-R1063H (Fig. 1I right). This result suggested that due to the decreased binding affinity with EpoR, the Jak2-R1063H signaling is slightly compromised but not physiologically manifested as anemia (in young animals) due to the compensation by slightly increased production of Epo. Vice versa, increased coupling of Jak2-R1063H with TpoR promotes mild thrombocytosis without elevated circulating Tpo.

### *Jak2*-R1063H alters normal hematopoietic development and causes premature HSC aging and functional decline of HSC compartment

**Fig. 2:**
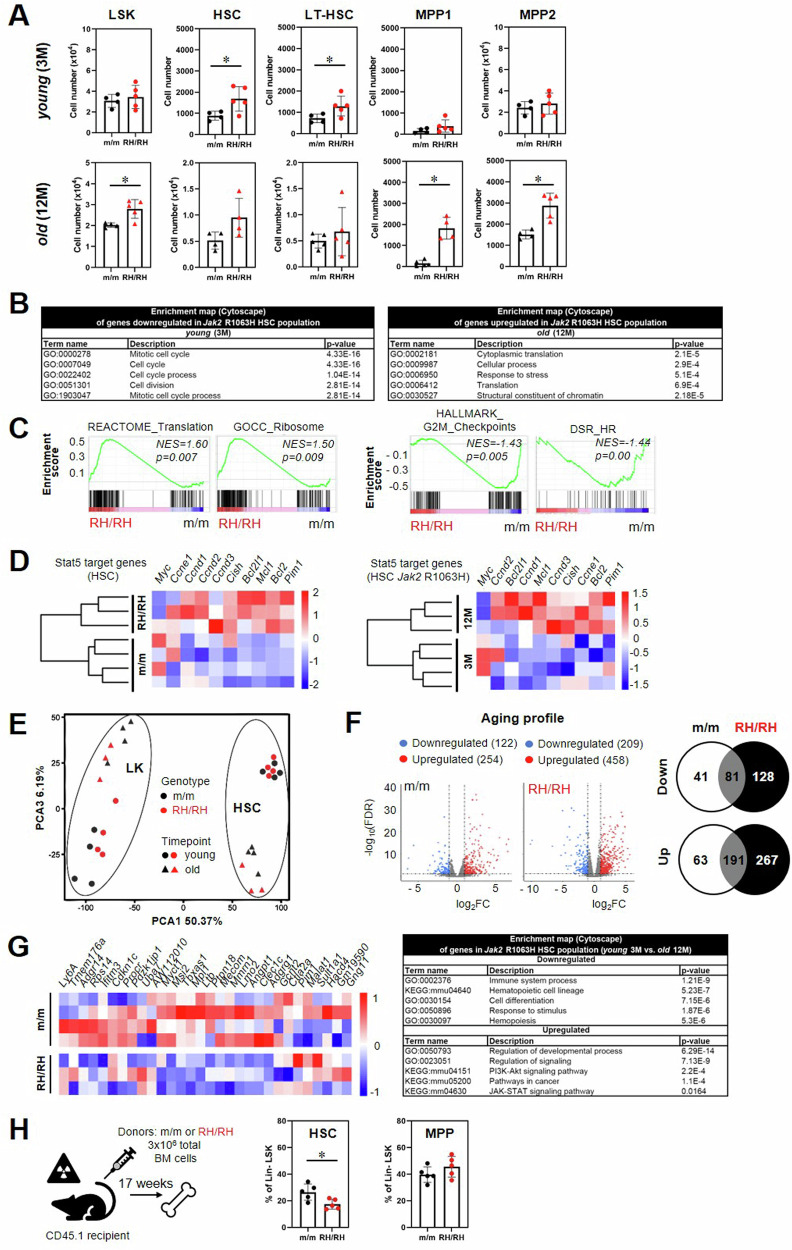
*Jak2*-R1063H mutation alters hematopoietic development and causes HSC functional decline with age. **A** Quantification of absolute number of distinct stem and progenitor populations in BM. Lin^−^ c-Kit^+^ Sca-1^+^ (LSK), Lin^−^ c-Kit^+^ Sca-1^+^ CD48^−^ CD150^+^ (HSC), Lin^−^ c-Kit^+^ Sca-1^+^ CD48^−^ CD150^+^ CD34^−^ CD135^−^(LT-HSC), Lin^−^ c-Kit^+^ Sca-1^+^ CD48^−^ CD150^+^ CD34^+^ CD135^-^ (MPP1), Lin^−^ c-Kit^+^ Sca-1^+^ CD48^+^ CD150^+^ CD34^+^ CD135^−^ (MPP2) (*n* ≥ 4 mice per group). **B** Relevant unique downregulated and upregulated GO terms in RH/RH HSCs, as calculated using Cytoscape platform (STRING App). **C** GSEA for transcriptional activation and HSC dysregulation of young (3 M) RH/RH vs. m/m, NOM *p* values < 0.05. **D** Hierarchical clustering of expression levels of Stat5 targets clearly distinguishes old *Jak*2-R1063H HSCs from wt (m/m) HSC (left) and from their young *Jak*2-R1063H counterparts (right). Heatmap representations of differential expression of Stat5 target genes. Red indicates upregulation, blue indicates downregulation of gene expression, and color intensity indicates the level of differential expression. Rows in the heatmaps represent individual samples. **E** Principal component analysis (PCA) (*n* = 4 mice per group, only RH/RH old mice (12 M) *n* = 3). **F** Volcano plot depicting expression levels of genes upregulated (red points) and downregulated (blue points) during aging (3 M versus 12 M) in m/m and RH/RH HSCs; FDR < 0.05, │log_2_FC│>1 (up left). Venn diagrams of intersecting upregulated or downregulated genes |log_2_FC |   ≥  2, *p*  <  0.05 comparing m/m and RH/RH HSCs during aging (up right). Relevant unique upregulated and downregulated GO terms and KEGG pathways in RH/RH HSCs, as calculated using Cytoscape platform (STRING App) (down). **G** Heatmap of top 25 genes representing mouse LT-HSC compartment. **H** Workflow of transplantation of unsorted BM cells to lethally irradiated recipients (*n* = 5 mice per group) and frequencies of BM HSC and MPP 17 weeks after transplantation. Data are presented as mean ± SD and unpaired t-test with Welsch’s correction was used for group comparison. *p < 0.05.

While the total number of LSK cells in the BM remained unchanged in the young animals, the *Jak2*-R1063H model displayed increased number of HSCs, specifically long-term hematopoietic stem cells (LT-HSCs) at a young age (3 M); the proportion of populations of short-term hematopoietic stem cells (MPP1) and multipotent progenitors (MPP2) remained unchanged at this stage (Fig. 2A). In aged mice (12 M), *Jak2*-R1063H mice exhibited increased frequency in the LSK population, mainly due to the increase in MPP1 and MPP2 (Fig. 2A). The levels of myeloid-biased multipotent progenitors (MPP3) and lymphoid-biased multipotent progenitors (MPP4) were not significantly altered (Supplemental Fig. 2).

Transcriptional analysis of HSCs sorted at 3 and 12 months of age revealed 54 genes and 124 genes, respectively, to be expressed differentially between the wt and *Jak2*-R1063H (i.e. differentially expressed genes (DEGs), FDR ≤ 0.05; Supplemental Table 1). Processes related to cell cycle were downregulated in the young mutant HSCs (Fig. 2B left). These data suggested accumulation of less cycling, relatively more quiescent cells in the young R1063H BM when compared to wt. Also examples of other significantly downregulated GSEA in the mutant 3-month-old HSCs (G2M checkpoint gene set, double strand repair by homologous recombination gene set, Fig. 2C) are consistent with more quiescent HSC phenotype [21–23]. These data indicated that downregulation of pathways related to cycling/cell cycle progression, known to be downregulated in old HSCs [21, 24], are consequences of premature aging of R1063H HSCs in 3-month-old mice. Also, significantly upregulated processes in the R1063H HSCs, translation and ribosomal protein genes expression (Fig. 2C left) and activated mTorc1 signaling (Supplemental Fig. 3A) are compatible with HSC aging [24, 25]. In the 12-month-old mice, lack of differential expression of the cell cycle-related gene sets likely reflects their comparative expression achieved by physiological aging of the wt HSC controls, but the processes associated with ribosomal genes expression remained enriched in the mutant HSCs of the 12-month-old animals (Fig. 2B right).

Then we questioned whether a significant increase of LSK, that is predominantly caused by significant increase of MPP1/MPP2 populations in aged R1063H murine BM (Fig. 2A), could result from a pro-proliferative signaling, known to be activated in MPP1/MPP2 cell subsets [26]. Earlier studies showed that loss of STAT5 expression in hematopoiesis specifically reduces numbers of MPP1/MPP2 [27], so opposite effect would be expected for augmented STAT5 activity. We assessed the expression of previously defined key STAT5 target genes in hematopoiesis [28] and found that their increased expression clearly distinguishes R1063H and wt HSCs in 12 M (Fig. 2D left). This differential expression was age-dependent, not present in 3-month-old HSCs, and distinguished also HSCs derived from young and old mutant mice (Fig. 2D right). These data suggested that the transcriptional differences in HSC compartment between the young and aged mutant animals, reinforced during aging, could be primarily linked to life-lasting chronic stress-induced conditions caused by the weakly activating *Jak2* mutation.

To better characterize the extent of intrinsic R1063H HSCs alterations during aging, we constructed the principal component analysis and identified age as the main source of transcriptional variation in HSC and also LK compartment (Fig. 2E). The skewing of transcriptional activation of aged HSCs was more pronounced in *Jak2*-R1063H population (Fig. 2F). Analysis of DEGs during aging confirmed, besides the mentioned JAK/STAT activation, also upregulation of PI3K-Akt signaling and the down-regulation or loss of immune system process and hematopoietic differentiation (Fig. 2F). These data likely reflect a known phenomenon of diminished differentiation and impaired immune response associated with a decrease of lymphoid-biased HSCs in aged hematopoiesis [24, 29, 30]. HSC compartment at 12 M also showed downregulation of majority of LT-HSC specific genes [31] in *Jak2-*R1063H cells, confirming faster, premature functional decline and relative exhaustion of this cell population in mutant animals (Fig. 2G). Using publicly available RNA-seq datasets (GSE123401) [32], we show that the transcriptional features of exhaustion/premature aging of *Jak2*-R1063H HSCs in BM of young mice are comparable to those that characterize HSC of young mice carrying Vav-Cre *Jak2*-V617F (see Supplemental Methods and Supplemental Fig. 3B–D).

Next, we aimed to determine whether the observed less cycling, more quiescent HSC status in young *Jak2*-R1063H mice is intrinsically driven and maintained after transplantation into wt recipients [33, 34]. We non-competitively transplanted total BM derived from control and R1063H littermates into CD45.1 recipients and assessed HSC numbers 17 weeks post-transplantation. The frequency for *Jak2*-R1063H HSCs was decreased compared to wt control mice, while the MPP frequencies were comparable (Fig. 2H). These results support our findings that *Jak2*-R1063H mutation results, at least in part, in HSC-autonomous premature aging/functional decline, but do not exclude microenvironmental contribution (driven by cell-extrinsic factors present in the *Jak2*-R1063H BM niche) to the pathological premature aging and functional decline of hematopoiesis in *Jak2*-R1063H mice.

### Myeloid bias, enhanced megakaryopoiesis and inflammatory signatures in *Jak2*-R1063H mutant hematopoiesis

**Fig. 3:**
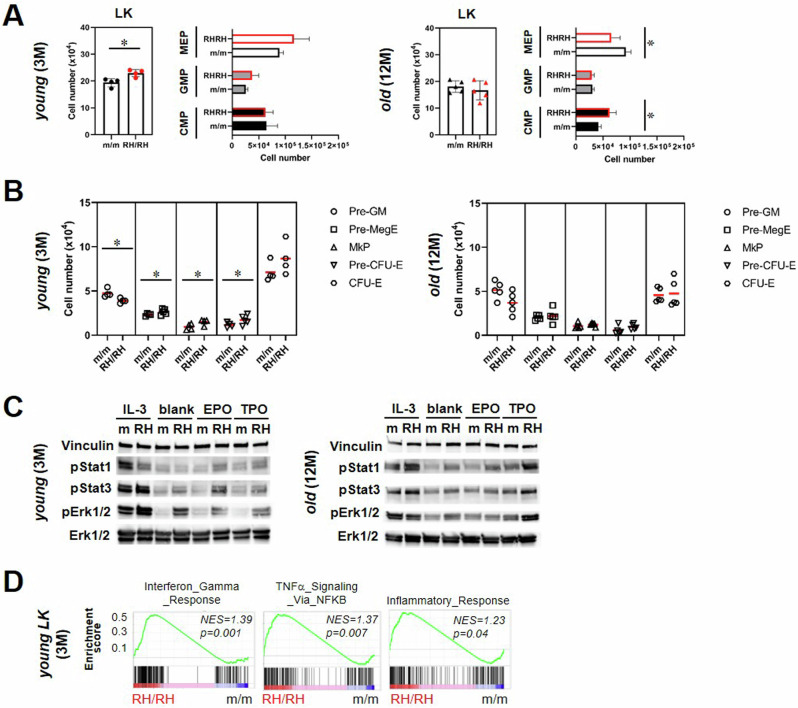
Inflammatory signaling and myeloid skewing in *Jak2*-R1063H mutant hematopoiesis. **A** Quantification of absolute number of myeloid precursors in BM. Lin^−^ c-Kit^+^ Sca-1^−^ (LK), Lin^−^ c-Kit^+^ Sca-1^−^ CD34^+^ FcgRII/III^low^ (CMP), Lin^−^ c-Kit^+^ Sca-1^−^ CD34^+^ FcgRII/III^+^(GMP) and Lin^−^c-Kit^+^ Sca-1^-^ CD34^-^ FcgRII/III^low^ (MEP) (*n* ≥ 4 mice per group). **B** Quantification of absolute number of erythroid/megakaryocytic committed subsets - Lin^−^ c-Kit^+^Sca-1^−^/CD41^−^/CD150^low^/CD105^+^ (CFU-E), Lin^−^c-Kit^+^Sca-1^−^/CD41^−^/ CD 150^+^/CD105^+^ (Pre–erythroid CFU), Lin^−^c-Kit^+^Sca-1^−^/CD41^+^/CD150^+^ (MkP), Lin^−^c-Kit^+^Sca-1^−^/ CD41^low^ /CD150^+^/CD105^−^ (Pre-MegE) and Lin^−^c-Kit^+^Sca-1^−^/CD41^low^/CD150^−^/CD105^-^ (Pre-GM) (*n* ≥ 4 mice per group). **C** Phosphorylation of Stat1 (Tyr 694), Stat3 (Tyr 705) and Erk1/2 (Thr 202/Tyr 204) proteins in m/m (wt) and RH/RH c-Kit^+^ enriched fraction of total BM. Total Erk protein and Vinculin served as loading control. WB shows the baseline (blank line) and cytokine-stimulated protein activities in protein lysates from enriched c-kit^+^ cells, starved for 40 hours and unstimulated or stimulated with indicated cytokines for 15 min. **D** GSEA for selected pathways from Hallmark gene sets enriched in RH/RH vs. m/m in LK cells, NOM *p* values < 0.05. Data are presented as mean ± SD and unpaired t-test with Welsch’s correction was used for group comparison. *p < 0.05.

Based on the above data (Fig. 1D), we reasoned that the direct pathway of HSC differentiation to MkPs is promoted by *Jak2*-R1063H in aged mice. We questioned if the canonical megakaryopoiesis pathway, which functions in parallel with the non-hierarchical pathway [19], is also enhanced and whether it may function more prominently at younger age. To explore the functional consequences of *Jak2*-R1063H expression in the myeloid progenitor compartment further, and in view of described myeloid bias and augmented megakaryocyte expansion associated with hematopoietic aging [34, 35], we analyzed immunophenotypically-defined LK cells (Lin^−^/Sca1^−^/cKit^high^) in the *Jak2*-R1063H murine BM. We observed non-significant increase in megakaryocytic/erythroid progenitors (MEP) at 3 months of age (Fig. 3A, left), and substantial myeloid bias in aged mice, characterized by a disproportionate increase in CMP cells and a marked decrease in MEP cells (Fig. 3A, right). At the level of committed cell subsets downstream of MEP, we observed significantly increased megakaryocytic and erythroid progenitors in young R1063H mice (Fig. 3B left); the trend towards expansion of pre-CFU-Es and megakaryocyte progenitor skewing persisted also in old mice (Fig. 3B, left).

We questioned whether megakaryocytic differentiation program (Figs. 1D and 3B) and thrombopoiesis in aged *Jak2*-R1063H mice (Fig. 1C) could be explained on the level of differential STAT activation downstream *Jak2*-R1063H mutation. It was previously reported that in *JAK2*-V617F CD34^+^ progenitors, intrinsically increased STAT1 phosphorylation promoted enhanced megakaryocytic and reduced erythroid differentiation and ET-like phenotype [36]. To evaluate differences in signaling caused by Jak2-R1063H kinase, we examined the phosphorylation levels of Stat1, Stat3, and Erk1/2 within the c-kit^+^-enriched BM population. WB analysis revealed increased baseline Stat1 phosphorylation level for *Jak2*-R1063H cells, with a marked increase after the short pulse stimulation with IL3, EPO and also TPO in old mice (Fig. 3C right); in the young *Jak2*-R1063H mice, the activation could be better demonstrated for Stat3 (Fig. 3C left). Cell-intrinsic inflammatory properties were demonstrated for *JAK2*-V617F myeloid progenitors [37, 38]. To test this aspect, we could only directly compare (at the gene expression level) LK cells from the 3-month-old mice, where individual myeloid populations did not differ significantly between mutant and wt animals (Fig. 3A). We observed interferon gamma response, TNFα signaling via NF-κB and inflammatory response gene sets significantly enriched in the *Jak2*-R1063H mutant LKs (Fig. 3D). These data are consistent with chronic inflammatory signaling mediated by *Jak2*-R1063H in the mouse BM compartment.

### Gene expression profiles suggest a key role for *Egr1* in transcriptional regulatory network of aged *Jak2*-R1063H progenitors

**Fig. 4:**
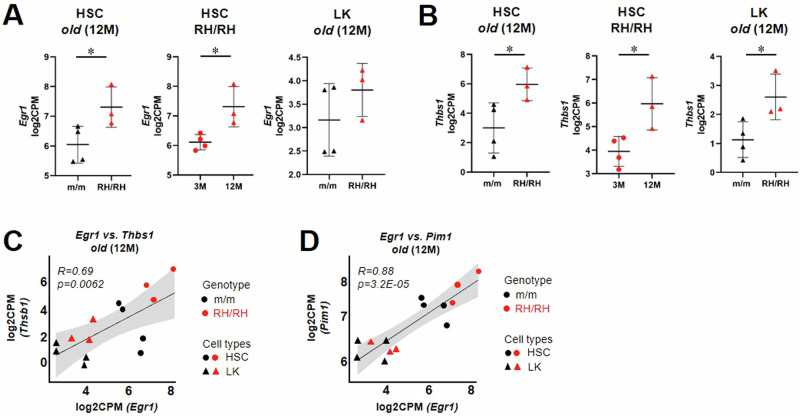
Egr1-mediated transcriptional reprogramming in aged *Jak2-*R1063H HSCs. **A**, **B** Differential expression of *Egr1* (**A**) and *Thbs1* (**B**) genes in HSC and LK compartments of *Jak2-*R1063H (RH/RH) and wt (m/m) animals. Boxplots generated from RNA-seq data. Student’s *t* test with Bonferroni correction, *p < 0.05; CPM counts per million. **C**, **D** Correlation curves depicting a significant strong positive correlation of *Egr1* and *Thbs1* expression levels (**C**) and a significant very strong positive coregulation of *Egr1* and *Pim1* (**D**) genes in HSC and LK compartments. Pearson correlation analysis combined values of both wt (m/m, black dots) and *Jak2-*R1063H (RH/RH, red dots). CPM counts per million, *p* value, *R* correlation coefficient.

Among the genes differentially overexpressed across the HSC/progenitor *Jak2*-R1063H populations, we identified an immediate early response gene *Egr1* (Supplemental Table 1), a known transcriptional master regulator of HSC aging, quiescence and overall HSC/progenitor functionality [39, 40]. *Egr1* was significantly upregulated in the *Jak2*-R1063H 12 M HSCs vs. wt 12 M HSCs and also in the *Jak2*-R1063H 12 M HSCs vs. 3 M mutant HSCs (Fig. 4A). In addition, although increased *Egr1* transcript levels in *Jak2*-R1063H 12 M LK cells vs. wt 12 M LK cells did not reach significance (Fig. 4A), some of the key Egr1 targets, as mentioned bellow, were significantly upregulated in both HSC/LK mutant populations. We searched for overlap between the putative EGR1 target genes in the ChIP-seq datasets (ENCODE transcription factor dataset) [41], and genes differentially upregulated in *Jak2*-R1063H 12 M HSCs vs. 3 M HSCs (Supplemental Table 1) and found that many of upregulated genes in 12-month-old mutant HSCs are targets of EGR1; many of these target genes were also upregulated in 12-month-old R1063H HSCs vs. 12-month-old wt HSCs. These data suggested a progressive augmentation of a subset of *Egr1* transcriptional signature in the *Jak2*-R1063H HSCs/progenitors.

Among the genes functionally validated as responsible for HSC aging [42], *JUNB, ZFP36, IER2* or *Cited2*, are proposed EGR1 targets in biomedical database [41] and showed different expression pattern (Supplemental Table 1). The most differentially upregulated *Egr1* target in 12 M mutant HSCs compared to wt (Supplemental Table 1) was *Thbs1*, the gene encoding thrombospondin 1. *Thbs1* was also significantly upregulated in 12 M mutant LKs and HSCs vs. 3 M mutant HSCs (Fig. 4B). *Thbs1* is a ligand for CD36 and CD47 receptors and it is implicated in angiogenesis, tumor growth and thromboembolism [43, 44]. Correlation analysis [45] revealed a strong positive correlation of *Egr1* expression with the *Thbs1* expression in HSC/LK populations, suggesting their functional relationship in these cells (Fig. 4C). One of the differentially upregulated Stat5-target genes was *Pim1* (Fig. 2D), that is commonly found upregulated in *JAK2*-V617F polycythemia vera (PV) and other myeloid malignancies [46]. Pim kinases are considered to be “weak oncogenes” and it seems that upregulation of this gene alone is not sufficient to promote overt disease such as PV or AML [46, 47], but may cooperate in its development. *Egr1* expression and *Pim1* expression revealed a very strong positive coregulation in HSC/LK populations in 12 M mice (Fig. 4D), but such a possible relationship in *Jak2*-R1063H progenitors requires further functional analyses.

### *Jak2*-R1063H mutation represents risk factor for thrombosis

**Fig. 5:**
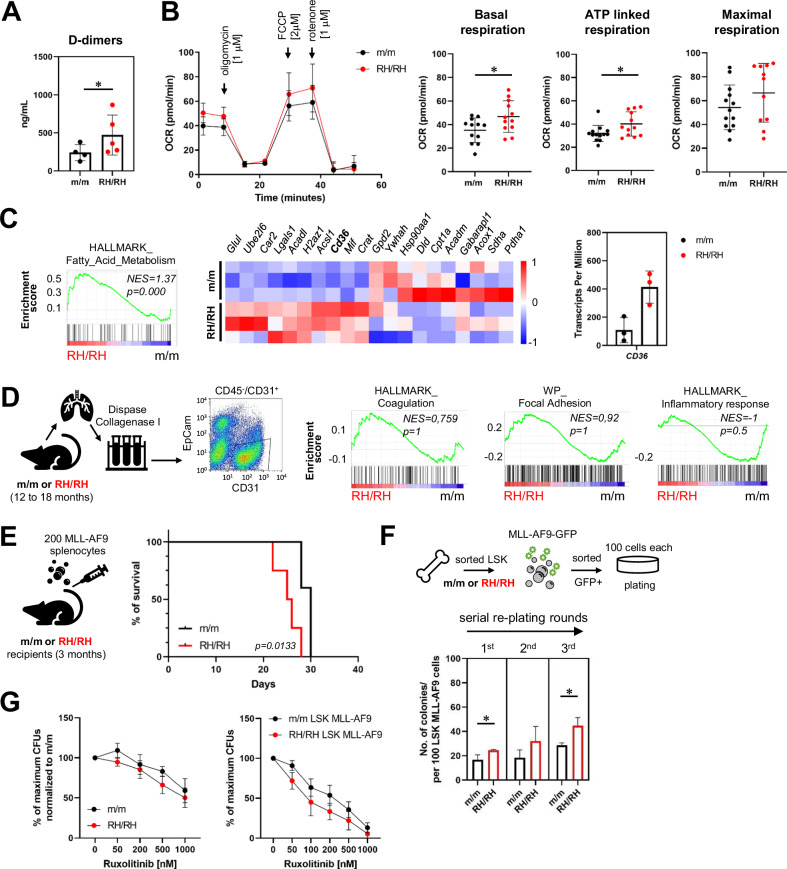
*Jak2*-R1063H mutation represents increased risk for thrombosis with altered bioenergetic PLT metabolism and accelerates leukemia transformation. **A** D-dimers concentration in plasma (*n* ≥ 4 mice per group). **B** Combined PLTs OCR profiles from m/m (wt) and RH/RH mice (young (3 M)). Quantification of basal, ATP linked and maximal respiration are shown in right (*n* ≥ 6 mice per group). **C** GSEA enrichment plots for Hallmark “Fatty acid metabolism” gene set enriched in RH/RH vs. m/m (wt) PLTs. Heatmap of top ten most upregulated and downregulated genes from Hallmark “Fatty acid metabolism” gene set (*n* = 3 mice per group, middle). Transcripts per million of *CD36* gene in PLT from m/m and RH/RH mice (young (3 M), right). **D** Workflow of pulmonary ECs isolation and representative flow cytometry plot (left). GSEA for selected pathways enriched in *Jak2*-V617F ECs [50] that are not over-represented in *Jak2*-R1063H mice (right). **E** Workflow. Kaplan-Meier survival analysis of m/m (*n* = 6) and RH/RH (*n* = 5) mice intravenously injected with 200 MLL-AF9 leukemic cells. MLL-AF9 splenocytes isolated from a leukemic mouse were transplanted into non-irradiated 10- to 14- weeks old m/m (wt) and RH/RH recipients. Mantle-Cox test was used to assess statistical significance of obtained results (*p* = 0.0133). **F** Workflow. GFP sorted 100 LSK cells transduced with MLL-AF9 oncogene were plated in semisolid media and total number of colonies were evaluated after 7 days. For serial re-plating, colonies were harvested from the methylcellulose media, washed, counted and seeded again. **G** Analysis of colonies grown in semisolid media with the increasing concentration of ruxolitinib, when Y-axis indicates the proportion of colonies to absolute numbers (*n* = 3 mice per group (left), 3 independent experiments in right). Data are presented as mean ± SD and unpaired t-test with Welsch’s correction was used for group comparison. *p < 0.05.

Our previous analysis of MPN patients bearing concomitantly *JAK2*-R1063H and V617F mutations revealed increased incidence of thrombotic events [14], so we asked whether the increased mortality in the cohort of *Jak2*-R1063H mice (which occurred suddenly precluding pathological analysis) (Fig. 1A) could be related to platelet pathology, similar to that observed for *JAK2*-V617F [17, 48]. We measured the levels of D-dimers as a marker of thrombosis and found them significantly increased in the young *Jak2*-R1063H group (Fig. 5A). Using Seahorse flux analyzer, we measured extracellular acidification rate (ECAR) that reflects cellular glycolysis, and oxygen consumption rate (OCR) as a measure of mitochondrial metabolism. We showed significant increase in basal/ATP-linked respiration, while maximal respiration remained unchanged (Fig. 5B), suggesting higher utilization of mitochondrial ATP by *Jak2*-R1063H PLTs (Supplemental Fig. 4). In subsequent RNA-seq analysis we demonstrated strong enrichment of fatty acid metabolism genes, including type II scavenger receptor CD36 (Fig. 5C), which was shown to promote PLT activation and thrombosis by activating redox sensing/signaling events [49]. As mentioned above, the most differentially upregulated gene in 12 M mutant HSCs compared to wt was *Thbs1*, the gene encoding thrombospondin 1, a CD36 ligand (Fig. 4B). Also, as noted above, the hyperactive PLTs produced by the platelet-biased HSC trajectory are prone to thrombosis [19]. Overall, the presence of the germline *Jak2-*R1063H mutation leads to a significant increase in PLTs in mice (Fig. 1C), independent of Tpo levels (Fig. 1D), and to increased thrombotic markers (Fig. 5A).

Endothelial cells (ECs) play a critical role in maintaining vascular homeostasis and regulating thrombotic responses. In the context of *JAK2*-V617F-driven MPNs, ECs harboring the mutation have been shown to adopt a pro-inflammatory, pro-adhesive, and pro-thrombotic phenotype, contributing to the higher incidence of vascular events in these patients [50, 51]. Nevertheless, analysis of pulmonary ECs in the *Jak2*-R1063H model did not reveal similar features (Fig. 5D), thus does not provide evidence of the link between ECs and *Jak2*-R1063H-associated pathobiology.

### Cooperative role of *Jak2*-R1063H mutation in leukemic development

The presence of *JAK2-*R1063H has been proposed as a potential risk factor for progression from MPN to AML [9]. In order to mimic leukemic transformation, we used model based on overexpression of the oncogenic fusion protein MLL-AF9 [52]. MLL-AF9 splenocytes were transplanted into wt or mutant mice, allowing us to determine the effects of the mutant niche on leukemic progression. We observed quicker leukemic onset in *Jak2*-R1063H than in wt mice (Fig. 5E, Supplemental Fig. 5) suggesting cell extrinsic effect of *Jak2*-R1063H mutation which might favor leukemogenesis. In addition, *Jak2*-R1063H HSCs transduced with MLL-AF9 oncogene exhibited both enhanced colony-forming potential upon MLL-AF9 introduction and increased re-plating capacity, confirming also the intrinsic effect of *Jak2*-R1063H mutation in promoting leukemic development (Fig. 5F). A previous in vitro sensitivity assay of Ba-F3/EPOR cells showed that human *JAK2*-R1063H and *JAK2*-V617F/R1063H double mutant cells are significantly more sensitive to JAK2 inhibitor, ruxolitinib, compared to wt cells [14]. The colony assay of wt and *Jak2*-R1063H BM cells under increasing concentration of ruxolitinib showed similar results. The consistent result was observed also by cultivating wt and *Jak2*-R1063H LSKs transduced with MLL-AF9, suggesting potential therapeutic implication (Fig. 5G). Structural modeling of interactions in the JAK2 wt and the R1063H mutant active site with ruxolitinib suggested stronger binding into the mutant (Supplemental Fig. 6). These findings provide a plausible structural explanation for the increased sensitivity of the R1063H mutant to JAK2 inhibition by ruxolitinib.

### Clinical outcomes of MPN patients carrying the *JAK2*-R1063H germline variant support that it is a risk-conferring variant

**Fig. 6:**
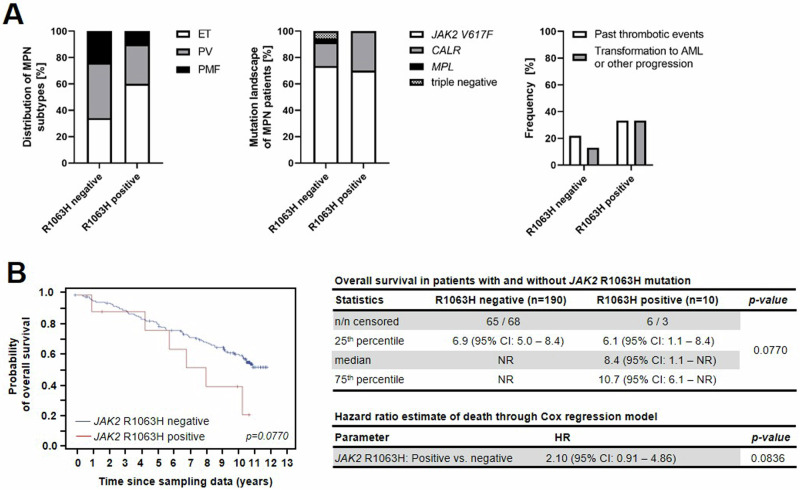
Clinical data. **A** Descriptive characteristics of MPN patients negative and positive for the *JAK2*-R1063H variant. Left graph shows higher proportion of ET in the *JAK2*-R1063H-positive group. Middle graph shows mutational landscape within the *JAK2*-R1063H-negative and R1063H-positive patient groups. Right graph shows the rate of thrombotic events and disease progression (transformation and post-PV fibrotic progression) in the *JAK2*-R1063H-negative and R1063H-positive group of patients. None of the patients who progressed in the *JAK2*-R1063H-positive group had a mutation in the *TP53* gene; 4 patients with *TP53* mutation were among the 19 patients who progressed in the *JAK2*-R1063H-negative group. **B** Kaplan-Meier curve of OS of MPN patients negative and positive for the *JAK2*-R1063H variant (left). Patients with the *JAK2*-R1063H mutation who progressed to sAML (*n* = 3) were treated with hydroxyurea (HU) (*n* = 2) and one patient received HU plus interferon. For patients with the *JAK2*-R1063H mutation who did not progress (*n* = 7), treatment included HU (*n* = 2), anagrelide (*n* = 3), or a combination of HU and anagrelide (*n* = 2). Kaplan-Meier estimate of OS in patients with and without *JAK2*-R1063H mutation (right upper panel). CI confidence interval, n/n censored = number of dead patients/number of censored patients, NR not reached. *p* value was obtained through statistical testing of OS with Log-rank test. 40 patients with missing data were not included in the survival analysis. For Cox regression model of hazard ratio estimate of death (right lower panel): CI confidence interval, HR hazard ratio. *p* value was obtained through Chi-squared test. 40 patients were excluded due to missing data. For additional clinical data see Supplemental Tables 2A, B.

Samples from 200 consecutive MPN patients (71 ET, 46 primary myelofibrosis (PMF), 83 PV) were analyzed for the presence of *JAK2*-R1063H variant. This variant was found in 10 patients (5%) – 6 ET (8.4% of all ET patients), 3 PV (3.6% of all PV patients) and 1 PMF (2.2% of all PMF patients) **(**Fig. 6A left). All *JAK2*-R1063H-positive MPN patients were also *JAK2*-V617F-positive, except for 3 *CALR*-positive ET patients (Fig. 6A middle). No significant difference was found between *JAK2*-R1063H positive and negative patients in terms of age at diagnosis (60 vs. 52 years), sex (female percentage 60% vs. 62%), and occurrence of bleeding (0 vs. 6%) (Supplemental Table 2A). However, there was a trend towards a higher incidence of thrombotic events (33% vs. 22%) and especially disease progression – i.e. transformation to AML or post-PV fibrotic progression (33% vs. 13%) in *JAK2*-R1063H-positive group (Fig. 6A right, Supplemental Table 2A). All progressions to AML were observed in patients with an initial diagnosis of ET. Moreover, patients with *JAK2-*R1063H had shorter median OS (8.4 years vs. not reached) (Fig. 6B). Comparison of the healthy Czech population from the 1000 Czech Genomes Project [53] with a group of our 200 MPN patients showed that the minor allele frequency (MAF) of the *JAK2*-R1063H in the healthy population is lower than in the group of MPN patients (1.67% vs. 2.50%; not statistically significant). The aggregate frequency of the *JAK2*-R1063H allele in general European population is much lower (0.58%) [54].

## Discussion

Because JAK2 activities and downstream signaling have pleiotropic effects in hematopoiesis [55, 56], we asked whether weakly activated Jak2, a lifelong consequence of the *JAK2*-R1063H variant, leads to the evolution of an age-associated phenotype.

In our mouse model, HSCs and progenitors expressing *Jak2*-R1063H variant revealed some characteristics of accelerated (premature) aging, including selected transcriptional characteristics [24, 25], myeloid-skewed differentiation [34] or impairment of HSCs [30] with observed loss of LT-HSC gene expression signature and decreased repopulation potential following transplantation [33, 34]. Increased anemia in aged R1063H mice would then also reflect more progressive aging-related changes in hematopoiesis in the mutant BM [35]. Physiologically, HSCs of 3-month-old mice exhibit a quiescent transcriptional signature and MPP1/MPP2 exhibit a cycling/proliferative signature [26]. The fact that the *Jak2*-R1063H-positive HSCs are at 3 months even more quiescent and accumulated in greater numbers at this age suggests that they have an increased proliferative history from early development, which has contributed to their accelerated aging and myeloid bias [57]. The exhaustion/premature aging of *Jak2*-R1063H HSCs belong to phenotypic features that share similarities with the oncogenic V617F mutation, as we documented by comparative analysis with the Vav-Cre *Jak2*-V617F mouse model [32]. The most striking difference in the hematopoietic phenotype of the two mutations is the absence of granulocytosis in our model. Even though we have previously described statistically higher WBC and neutrophils in the double mutant *JAK2*-V617F/R1063H ET patients [14], the effect on granulocytosis in these patients could be caused by cumulative effect on JAK2 signaling of the two *JAK2* (germline R1063H and somatic V617F) mutations. Specific differences in granulopoiesis in humans and mice may also play a role [58].

Our data suggest that in *Jak2-*R1063H-positive hematopoiesis, cell-autonomous signaling with a likely contribution of a pro-inflammatory milieu promotes pro-thrombotic complications and risk for malignant development. Our data revealed intrinsic activation of Jak2/Stat5 signaling in the BM of young *Jak2*-R1063H-mutant mice and augmented activation of a set of Stat5 target genes in old animals’ BM cells. We also observed differential Stat3 and Stat1 activation in *Jak2*-R1063H-positive c-kit progenitors, with more exhibited Stat1 activation in aged mutant progenitors. In agreement with the *Jak2-*R1063H mouse phenotype, STAT1 is known to support megakaryocytic differentiation and to create a bias towards an ET phenotype [36]. Importantly, we observed age-dependent alterations in the activation of two distinct pathways for megakaryopoiesis, which give rise to PLTs [18, 19]. The traditional “myeloid bias” (which includes increased MkPs) was detectable in both, 3 M and 12 M old mutant mice, whereas accumulated stress (marked by Egr1 expression signature) appears to lead to an augmented direct MkP differentiation trajectory from platelet-biased HSCs only during aging. Further studies will be needed to clarify the role of *JAK2*-R1063H (as well as the role of other germline mutations associated with thrombocytosis) [10–12], in the regulation of both pathways.

In previous studies, chronic inflammatory conditions and *Egr1* overexpression have been connected with acquisition of molecular/phenotypical markers of aged-state of hematopoiesis [59, 60]. A key factor modulating the phenotype of *Jak2*-R1063H mice appears to be the overexpression of *Egr1* in aged HSCs/progenitors. Given that the expression of *Egr1* (and Egr1 targets) increases with animal age and that STAT and EGR1 signaling are directly or indirectly interconnected [61–63], we conclude that stress/inflammatory response-associated Egr1 signaling in aging mice likely reinforces some alterations initiated by Jak2/Stat target genes’ expression.

Patients with MPNs are known to be at an elevated risk of thrombosis, but the mechanism for MPN-related coagulation activation is not yet fully characterized [16]; there is evidence that *JAK2*-mutated ECs may contribute to the MPN pro-thrombotic state [45, 51]. We observed increased mortality in *Jak2*-R1063H mouse cohort and elevated levels of D-dimers, indicative of thrombotic complications, but the ECs from these mice did not exhibit pro-adherent and pro-thrombotic features. Whereas MPN patients typically exhibit activated OXPHOS and mTORC1 signaling pathways and enhanced mitochondrial activities in PLTs, both leading to PLT hyperactivity [17], *Jak2*-R1063H-positive PLTs metabolic abnormalities were associated with fatty acid metabolism. This metabolic shift is critical, since it was shown that mitochondrial ATP, needed for PLT granule secretion and thrombus formation, is primarily derived from fatty acid oxidation [64]. Thrombosis in MPN patients is a multifactorial event involving chronic inflammation [65], functionally hyper-reactive PLTs produced by direct megakaryocyte differentiation pathway from platelet-biased HSCs [66] and other complex interactions among various blood cells [16], so role of these processes and cell types in the thrombosis risk caused by the *Jak2*-R1063H mutation cannot be excluded. Differential overexpression of *Thbs1*, coregulated with *Egr1* in the mutant HSCs and LKs, may be involved in chronic inflammatory responses within the progenitors and promote the observed aging-related HSC defects [67]. Whether and how is thrombospondin 1 secreted and promotes the observed PLT activation and contributes to thrombosis remains to be determined.

In this study, we focused primarily on the cell intrinsic alterations caused by *Jak2-*R1063H that underlie the observed murine phenotype. The exception was the assessment of the effect of *Jak2*-R1063H on leukemia progression, in which we demonstrated both, cell-intrinsic as well as cell-extrinsic effect of the mutation on leukemia progression. The cell-autonomous factor contributing to the accelerated leukemic development could be overexpression of *Pim1* oncogene [46]. Pim1 expression was shown to be significantly increased in hematopoietic progenitors of *Jak2*-V617F knock-in mice [47] and was overexpressed in *JAK2*-V617F-positive ET patients [36, 68]. Whether a possible inflammatory microenvironment (mediated by R1063H-positive hematopoietic cells and nonhematopoietic niche cells) produces inflammatory factors that can act as the cell-extrinsic factors supporting the expansion of MLL-AF9 leukemic cells [69] remains undefined and should be further addressed by future research endeavors.

Earlier clinical studies have suggested a higher risk of thrombotic events in patients with *JAK2*-R1063H variant [14, 15] as well as a higher risk of transformation to AML [9], and our clinical evidence is consistent with these studies. Interestingly, all transformations to AML in our *JAK2-*R1063H-positive group occurred in patients initially diagnosed with ET; two of these ET patients carried the *CALR* driver mutation, none of them carried a mutation in the *TP53* gene. The frequency of the rare germline low-penetrance risk alleles predisposing to sporadic MPN may be population-specific [70]. The studied *JAK2* variant has a MAF of 1.67% in the Czech population, which is relatively high; the available database for *JAK2*-R1063H across multiple ethnicities reveals that the MAF is much lower for most populations [54]. Missing analyses of more diverse cohorts is a limitation of our study; further population studies will be needed to better delineate the overall disease risk conferred by this variant.

## Supplementary information

Supplemental Methods

Supplemental Figures

Supplemental Western Blots

Data Set 1

Data Set 2

## Data Availability

RNA-seq data are available in the BioStudies database (http://www.ebi.ac.uk/biostudies) under accession number S-BSST12345, E-MTAB-14686, E-MTAB-14685 and E-MTAB-15355.
