# Supplementary material for: Germline Jak2-R1063H mutation interferes with normal hematopoietic development and increases risk of thrombosis and leukemic transformation
Source: Leukemia. 2025 Aug 21;39(11):2745–57. doi: 10.1038/s41375-025-02737-w (PMC12589134; doi:10.1038/s41375-025-02737-w)
Supplement: Supplementary file 1 — Supplemental Methods [file 41375_2025_2737_MOESM1_ESM.pdf]

Title: Germline *Jak2*-R1063H mutation interferes with normal hematopoietic development and increases risk of thrombosis and leukemic transformation

Supplemental Methods

Mice

Murine blood samples were obtained by bleeding from the cheek or retroorbital sinus. Blood was analyzed with an BC-5300 or BC-30 Auto Hematology Analyzer (Mindray). Mice were sacrificed by cervical dislocation, femurs and tibias were isolated, and crunched using a pestle and mortar. Where indicated, mice were exposed to lethal (6 Gy) dose of the whole-body irradiation using Precision X-RAD 225XL equipped with Cu filter (0.5 mm).

RNA Sequencing

RNA sequencing and analysis of HSCs (defined as Lin^-, $Sca1^+/ckit^{high}$, $CD150^{high}/CD48^{low}$ cells), LK (defined as Lin^-, $Sca1^-/ckit^{high}$) cells and lung endothelial cells (defined as $CD45^-/CD326^-/CD31^+$ cells) sorted from young and/or old wt and *Jak2*-R1063H mice ($n = 3$-4) using Influx instrument was performed. RNA from sorted HSCs, LK cells, purified platelets and endothelial cells was extracted with the RNAeasy Micro Kit (Qiagen) and cDNA was synthesized using the SMARTer Stranded Total RNA-Seq Kit v2 Pico (Takara Bio) according to the manufacturer's instructions. Sequencing was performed by NextSeq 550 system (Illumina) using NextSeq 1000/2000 P2 or P3 XLEAP-SBS™ Reagent. Sequences were mapped to GRCm39 reference genome by STAR v2.7.9 software. Differential expression and normalized counts were determined by DESeq2 package in R software. Genes with expression change higher than $\log_2$FoldChange > 1 and p-adjusted value < 0.05 were considered as significantly up- or down-regulated.

Gene Set Enrichment Analysis

Gene set enrichment analysis (GSEA) was performed as previously described (1). Supporting data are available in the BioStudies database (http://www.ebi.ac.uk/biostudies) under accession number S-BSST12345, E-MTAB-14686 - Expression profiling of hematopoietic stem cells/progenitors from young (3 months) and old (12 months) homozygous *Jak2*-R1063H mice and wild-type controls, E-MTAB-14685 - Expression profiling of platelets from young (3 months) homozygous *Jak2*-R1063H mice and wild-type controls and E-MTAB-15355 - Expression profiling of pulmonary endothelial cells from homozygous *Jak2*-R1063H mice and wild-type controls.

Re-analysis of Datasets from Publicly Available Gene Expression Omnibus Database

Raw data from the project GSE123401 in FASTQ format were trimmed using Trimmomatic v0.39 in single-end configuration. Trimmed reads were then mapped to reference genome GRCm39 (mm39) using STAR v2.7.9. Resulting reads in BAM format were indexed and gene counting was performed using StringTie v2.2.1. Raw gene counts were subsequently filtered and normalized in R software v4.3.2 using edgeR package. GSEA analysis was then performed on normalized gene counts against chosen library of genesets.

Flow Cytometry

For flow cytometric analysis, single cell suspensions of bone marrow (BM) and spleen from wild-type (wt) and *Jak2*-R1063H mice were subjected to red cell lysis. Cells were depleted with EasySep™ Mouse Hematopoietic Progenitor Cell Isolation Kit (StemCell Technologies), stained with fluorescently-labeled antibodies (**Supplemental Figure 7**) and analyzed using a FACSymphony (BD Biosciences) flow cytometer. Gating strategy for flow cytometry analysis of BM subpopulations is provided as **Supplemental Figure 7**. Sorting of BM HSCs, MPP and LK cells was performed by two subsequent steps. First, the Lin^+ fraction of the BM cells was labeled using biotinylated lineage markers CD45/B220 (RA3-6B2), CD3 (145-2c11), Ter119 (TER-119), Gr1 (RB6-8C5), and CD11b (M1/70). These cells were then

incubated with anti-biotin magnetic beads (Miltenyi Biotec) and were isolated using MACS separator. Second, the Lin^- fraction of the BM was labeled with the c-Kit PE (2B8), Sca-1 APC (E13-161.7), CD48 FITC (HM48-1), CD150 Pe-Cy7 (TC15-12F12.2) antibodies and with streptavidin-eFluor450. Cell suspensions were stained with Hoechst 33258 to exclude dead cells and HSCs were sorted using Influx instrument (BD Biosciences). Data were obtained using Diva software (BD Biosciences) and analyzed using FlowJo software (Tree Star Incorporation).

Immunoprecipitation
Mouse V5-tagged receptors (Epor, Tpor) and human HA-tagged receptors (EPOR, TPOR) expressing HEK293 cells were cultured in DMEM, 10% FBS and no additional cytokines. The cells were transfected with pCMV6-AC-IRES-GFP-Puro mouse Jak2 and human JAK2 variants using Lipofectamine 2000 (Thermo Fisher Scientific). 48 hours post transfection cells were lysed in buffer containing 1 mM EDTA, 150 mM NaCl, 50 mM Tris pH 7.5, 0.4% Triton X-100, 2 mM $CaCl_2$, 2 mM $MgCl_2$, 1 mM DTT, 10 mM β-glycerol phosphate, 1 mM NaF, 0.1 mM PMSF, 0.1 mM Na_3VO_4, 2 μg/mL Aprotinin, 10 μg/mL Leupeptin. For immunoprecipitation of mouse variants V5-Trap® Agarose or DYKDDDDK Fab-Trap™ Agarose (ChromoTek) were used according to the manufacturer’s instructions. For immunoprecipitation of human variants EZview™ Red ANTI-FLAG® M2 Affinity Gel (Sigma) was used according to the manufacturer’s instructions. For maximal specificity of human JAK2 variants elution, the 3X FLAG® Peptide (Sigma) was used.

Proteins were resolved on commercial gradient SDS-PAGE 4-15% acrylamide gel (GenScript) and transferred to nitrocellulose membrane (Thermo Fisher Scientific) using semi-dry blotter (BioRad) 2.5 mA/cm^2, 10 W, 20 V, 30 min. Mouse receptors were detected using V5-Probe mouse monoclonal antibody (Santa Cruz, sc-271944) and human receptors were detected using anti-HA rabbit monoclonal antibody (Cell Signaling, #3724). Mouse Jak2 and human JAK2 were detected using anti-FLAG rabbit monoclonal antibody (Cell Signaling, #14793). Total cell lysates (TCL) were immunoblotted alongside the immunoprecipitated samples, anti-V5, anti-HA and anti-FLAG antibodies used for the detection are listed above, and loading controls anti-PCNA (Abcam, #ab18197) and anti-CtBP (Santa Cruz, sc-17759) were used. Antibodies were visualized by chemiluminescent SuperSignal™ West Substrate (Thermo Fisher Scientific). Quantitation of HA and Flag signals was performed using densitometric analysis by ImageJ software according to the software manual.

Seahorse
For platelets (PLT) isolation, whole blood was collected to acid-citrate-dextrose tubes supplemented with 3 μM prostaglandin E1 (PGE), and PLT-rich plasma was collected by centrifugation. Then, PLT-rich plasma was diluted with Seahorse basal medium (D5030, Merck, 0.2% BSA, 2 mM L-glutamine, 1 mM sodium pyruvate, 10 mM glucose, pH 7.4) supplemented with PGE and centrifuged. Pelleted PLTs were subjected to red cell lysis and resuspended in the presence of anti-CD45 beads (Miltenyi Biotec) to remove residual leukocytes. Platelets were seeded at a density of $6x10^8$ platelets/well in Seahorse XF24 Analyzer plates (Agilent Technologies) coated with Cell-Tak (Corning) and spinned for 5 minutes at 250 g (no brake). The initial oxygen consumption rate (OCR) and extracellular acidification rate (ECAR) and after addition of 1 μM oligomycin, 2 μM FCCP, and mixture of 1 μM rotenone, 1 μg/mL antimycin A and 100 mM 2-deoxyglucose were recorded (2). The OCR and ECAR parameters were calculated according to Agilent protocol as differences between initial and non-mitochondrial respiration (basal respiration), initial and oligomycin respiration (ATP-linked respiration), FCCP and non-mitochondrial respiration (maximal respiration), initial ECAR and 2-deoxyglucose addition (glycolytic activity) and oligomycin and 2-deoxyglucose addition (glycolytic capacity).

Isolation of Pulmonary Endothelial Cells
Mice were euthanized, and the anterior thoracic wall was removed to expose the heart and lungs. Lungs were perfused through the heart with 3–5 mL of Hank’s Balanced Salt Solution (HBSS) containing

dispase (diluted 1:300). Perfusion was performed under pressure using a syringe and needle to flush blood into the liver, causing the lungs to visibly blanch. To facilitate drainage, the inferior vena cava was transected. The lungs and heart were excised together and rinsed in HBSS on a Petri dish. The heart was removed, and the lungs from each animal were evenly divided into three 2.0 mL microcentrifuge tubes. Each portion received 100 µL of serum-free medium containing dispase (1:300), collagenase I (2 mg/mL), and DNase I (1:1000). Tissue was minced finely using sharp scissors. The volume was brought to 1 mL with enzyme-containing medium and incubated at 37°C on a thermomixer at 800 rpm for 10 minutes. This step was repeated three times, with the final digestion lasting 20 minutes to ensure thorough tissue dissociation. After each digestion, the cell suspension was transferred to a tube containing cold medium supplemented with 10% FBS to inactivate enzymes. After the final digestion, all material was filtered through a 70 µm nylon mesh filter with medium containing 10% FBS. The filtered cell suspension was centrifuged at 500 × g for 10 minutes at 4°C. The supernatant was discarded, and the pellet was resuspended with 1× ACK lysis buffer to remove erythrocytes. The mixture was incubated for 5 minutes on ice, then washed with 10 mL of medium containing 10% FBS and centrifuged again at 500 × g for 5 minutes at 4°C. The cell pellet was resuspended in 10% FBS medium containing fluorescently conjugated antibodies: anti-mouse CD45-Pacific Blue (PB; Biolegend), CD31-PE (eBioscience), and EpCAM-APC (CD236; eBioscience), each at a final dilution of 1:400. Unstained and single-color controls were included. Cells were incubated on ice for 15–30 minutes. Following staining, cells were washed with 10 mL of 10% FBS medium and centrifuged again (500 × g, 5 minutes, 4°C). The pellet was resuspended in 200–500 µL of HBSS with 3% FBS. The $CD31^+$ population was isolated; $EpCAM^+$ cells served as epithelial controls.

Enzyme-Linked Immunosorbent Assay (ELISA)
The serum levels of Epo, Tpo and D-dimers of young and old mice were quantified according to the manufacturer's instructions for the Mouse Epo, Tpo Quantikine ELISA Kit (R&D Systems) and ELISA Kit for D-Dimer (D2D) (Cloud-Clone Corp.). Measurement of iron parameters (Bilirubin, Fe, Ferritin, Mg, Transferrin, UIBC) was performed as a custom service by the Czech Centre for Phenogenomics (BIOCEV/IMG) using Clinical Pathology Panel.

Western Blotting Analysis
BM was isolated from young and old wt and *Jak2*-R1063H mice and fraction of $c\text{-}Kit^+$ cells was enriched using MACS separator. Cells were starved for 48 hours; thereafter EPO (20 U/mL), TPO (100 ng/mL) and IL3 (10 ng/mL) were added for 15 minutes. For phospho-STAT5 detection in whole BM lysates, BM cells were isolated from young wt (n=2) and *Jak2*-R1063H (n=2) mice and pooled according to matching genotypes. Cells were starved for 4 hours, thereafter EPO (20 U/mL), TPO (100 ng/mL), G-CSF (50 ng/mL) and IL3 (10 ng/mL) were added for 10 minutes. Extracted cells were lysed (150 mM NaCl, 50 mM Tris pH 7.5, 0.4 % Triton-X, 2 mM $CaCl_2$, 2 mM $MgCl_2$, 1 mM EDTA and cocktail of protease inhibitors). Equivalent of 1.5 x 10^6 enriched $c\text{-}Kit^+$ cells and 7.0 x 10^6 BM cells were loaded per lane. Human erythroleukemia (HEL) cell line lysate was included to provide human control. Protein lysates were separated using gradient SDS-PAGE 4-15% acrylamide gel, transferred to nitrocellulose membrane, and stained with following antibodies: phospho-JAK2 (Y1007/1008) (#3776), JAK2 (#3230), phospho-STAT1 (Y701) (#9167), STAT1(#14994), phospho-STAT3 (Y705) (#9145), STAT3 (#12640), phospho-STAT5 (Y694) (#9351), STAT5 (#9363), phospho-p44/42 MAPK (Erk1/2, T202/Y204 #4370), p44/42 MAPK (Erk1/2 #4695) and β-actin (#4970) (all Cell Signaling) or vinculin (Merck #05-386) as a loading control.

Whole BM Transplantation
3x10^5 BM cells from 12-week-old wt and *Jak2*-R1063H were transplanted into lethally irradiated C57BL/6NCrl ($CD45.1^+$) recipient mice by tail vein injection. 4, 9 and 17 weeks after transplantation peripheral blood from recipient mice was analyzed using BC-30 Auto Hematology Analyzer (Mindray).

MLL-AF9-Induced Leukemia *in vivo*
200 MLL-AF9 splenocytes isolated from a leukemic mouse (for details see)(3) were transplanted into non-irradiated 10- to 13-week-old wt and *Jak2*-R1063H recipients. Sorted 100 or 1000 GFP$^+$ MLL-AF9-infected LSK from wt and *Jak2*-R1063H mice were transplanted into non-irradiated 12- to 14-week-old recipient C57BL/6NCrl mice (CD45.1$^+$). Leukemia development was assessed during the course of the experiment by flow cytometric analysis and the % of c-Kit$^+$ and GFP$^+$ cells in blood was determined.

Virus Production and BM Transduction
HEK293-T cells in a 15-cm dish were transfected with 10 µg of pMSCV MLL-AF9-IRES-GFP, 10 µg pCL-Eco Retroviral Packaging Vector using Lipofectamine 2000 (Thermo Fisher Scientific). After 4 hours, the medium was replaced with fresh growth medium (DMEM + 10% FBS). After 24 and 48 hours, retroviral supernatant was harvested and filtered through a 0.45-mm filter. LSK cells were sorted from wt and *Jak2*-R1063H mice and transduced with MLL-AF9 retrovirus for 4 hours and *in vitro* expanded in Stem SFEM I (StemCell Technologies) medium supplemented with 10 ng/mL IL3, 20 ng/mL IL6, 100 ng/mL SCF, 50 ng/mL TPO, 100 ng/mL Flt3. Then 100 GFP$^+$ MLL-AF9-infected cells were sorted and used for *in vitro* re-plating assay (M3434, StemCell Technologies) or for *in vitro* colony assay with increasing concentration of ruxolitinib (0 – 1000 nM).

RNA Isolation and Real-Time PCR analysis
RNA from liver was extracted using QIAshredder and Rneasy Mini Kit (Qiagen). One µg of RNA was reverse transcribed with SuperScript VILO Master Mix with ezDNase (Thermo Fisher Scientific) and q-PCR was performed using LightCycler 480 SYBR Green I Master and the primers, FmHamp 5´AAGCAGGGCAGACATTGCG 3´, 5´ RmHamp CAG GATGTGGCTCTAGGCTATGT 3´, FmBetaActin 5´ GATCTGGCACCACACCTTCT 3´, RmBetaActin 5´ GGGGTGTTGAAGGTCTCAAA 3´.

Clinical Data and Analysis
We analyzed 200 consecutive patients from University Hospital Brno with confirmed myeloproliferative neoplasia for *Jak2*-R1063H using the Sanger sequencing method. *TP53* status was assessed by targeted NGS of total leukocyte DNA (limit of detection of 0.1%). Informed consent was obtained before sample collection. Descriptive summary of patients' demographic and clinical characteristics was performed for both groups (*JAK2*-R1063H-positive vs. *JAK2*-R1063H-negative). For continuous variables, mean with standard deviation (SD) as well as median with lower (Q1) and upper quartile (Q3) were calculated. For description of categorical variables, absolute and relative frequencies (i.e. percentages) were used. To test differences between the two groups, we performed the two-sample t-test (normality assumption not rejected) or non-parametric Mann-Whitney U test (normality assumption rejected) for continuous variables and Pearson's chi-squared test of Fisher's exact test (if case of low expected counts) for categorical variables. The level of statistical significance 5% was used. Overall survival was estimated using the Kaplan-Meier survival method. Overall survival probabilities were displayed through the Kaplan-Meier curves. The differences between the probabilities were compared across the studied groups through the Log-rank test. Furthermore, we employed Cox regression models to estimate hazards ratios (HRs) for death in patients with *JAK2*-R1063H variant and without *JAK2*-R1063H variant. Besides the crude hazard ratio, we obtained adjusted version with the presence of congenital thrombophilia (yes vs. no), BMI over 30 (yes vs. no), smoking (yes vs. no) and hypertension (yes vs. no) as confounders. Estimated hazard ratios were displayed through the forest plot (not shown). We did not impute missing data in this analysis and performed an available-case analysis. Statistical software SAS (version 9.4) was used for the analyses.

Statistics
Statistical significance for indicated data sets was determined using unpaired 2-tailed Student t-test and p values <0.05 were considered as statistically significant. Scatter dot plots depict mean with error bars representing standard deviation (SD). Survival analysis was performed using the Kaplan-Meier

method and log-rank Mantel-Cox test was used to assess statistical significance of survival differences between experimental groups. The number of mice was chosen to ensure 90% power with a 5% error based on the observed standard deviation. The number of animals in each experiment is indicated in the figure legend.

Structural Modeling of JAK2 Variant

To investigate the structural basis for the enhanced sensitivity of the JAK2-R1063H mutant to ruxolitinib, we conducted comparative modelling of the wt and mutant (canonical V617F and/or R1063H mutant) human JAK2 kinase domains using AlphaFold 3.0. Full-length sequences of human JAK2-wt, JAK2-V617F, JAK2-R1063H and JAK2 V617F/R1063H mutants were used as inputs for AlphaFold 3.0 (DeepMind). Default AlphaFold 3.0 parameters were used, with multiple sequence alignment (MSA) generation enabled for optimal structure prediction. The resulting models were evaluated using the predicted local distance difference test (pLDDT) scores to assess structural confidence. Following structure generation, the best scored folds of the individual proteins were compared with each other. Then, we extracted the JH1 kinase domain from each model and aligned them to the co-crystal structure of wild-type JAK2 with ruxolitinib (PDB ID: 6VGL) using PyMOL v2.5 (Schrödinger LLC). This allowed for direct comparison of the ruxolitinib binding pocket geometry and residue positioning between the wild-type and mutants. Special attention was given to changes in cavity size, shape, and potential drug-contact residues. In the R1063H model, the ruxolitinib was positioned into the active site extracted from the original co-crystal (PDB ID: 6VGL) based on the sequence alignment, to assess the possible binding interactions. All findings were visualized using PyMOL and structural interpretations were based on existing literature on JAK2 domain function and ruxolitinib binding dynamics.

References

1. Berková L, Fazilaty H, Yang Q, Kubovčiak J, Stastna M, Hrckulak D, et al. Terminal differentiation of villus tip enterocytes is governed by distinct Tgfβ superfamily members. EMBO reports. 2023;24(9):e56454.
2. Pecina P, Pajuelo D, Cunatova K, Houstek, Mracek T. Cytochrome c oxidase subunit 4 isoform switch results in modulation of oxygen affinity. Biochimica et Biophysica Acta (BBA) - Bioenergetics. 2018;1859:e21.
3. Grusanovic S, Danek P, Kuzmina M, Adamcova MK, Burocziova M, Mikyskova R, et al. Chronic inflammation decreases HSC fitness by activating the druggable Jak/Stat3 signaling pathway. EMBO reports. 2023;24(1):e54729.
