# Supplementary material for: Germline Jak2-R1063H mutation interferes with normal hematopoietic development and increases risk of thrombosis and leukemic transformation
Source: Leukemia. 2025 Aug 21;39(11):2745–57. doi: 10.1038/s41375-025-02737-w (PMC12589134; doi:10.1038/s41375-025-02737-w)
Supplement: Supplementary file 2 — Supplemental Figures [file 41375_2025_2737_MOESM2_ESM.pdf]

Title: Germline *Jak2*-R1063H mutation interferes with normal hematopoietic development and increases risk of thrombosis and leukemic transformation

Supplemental Figures

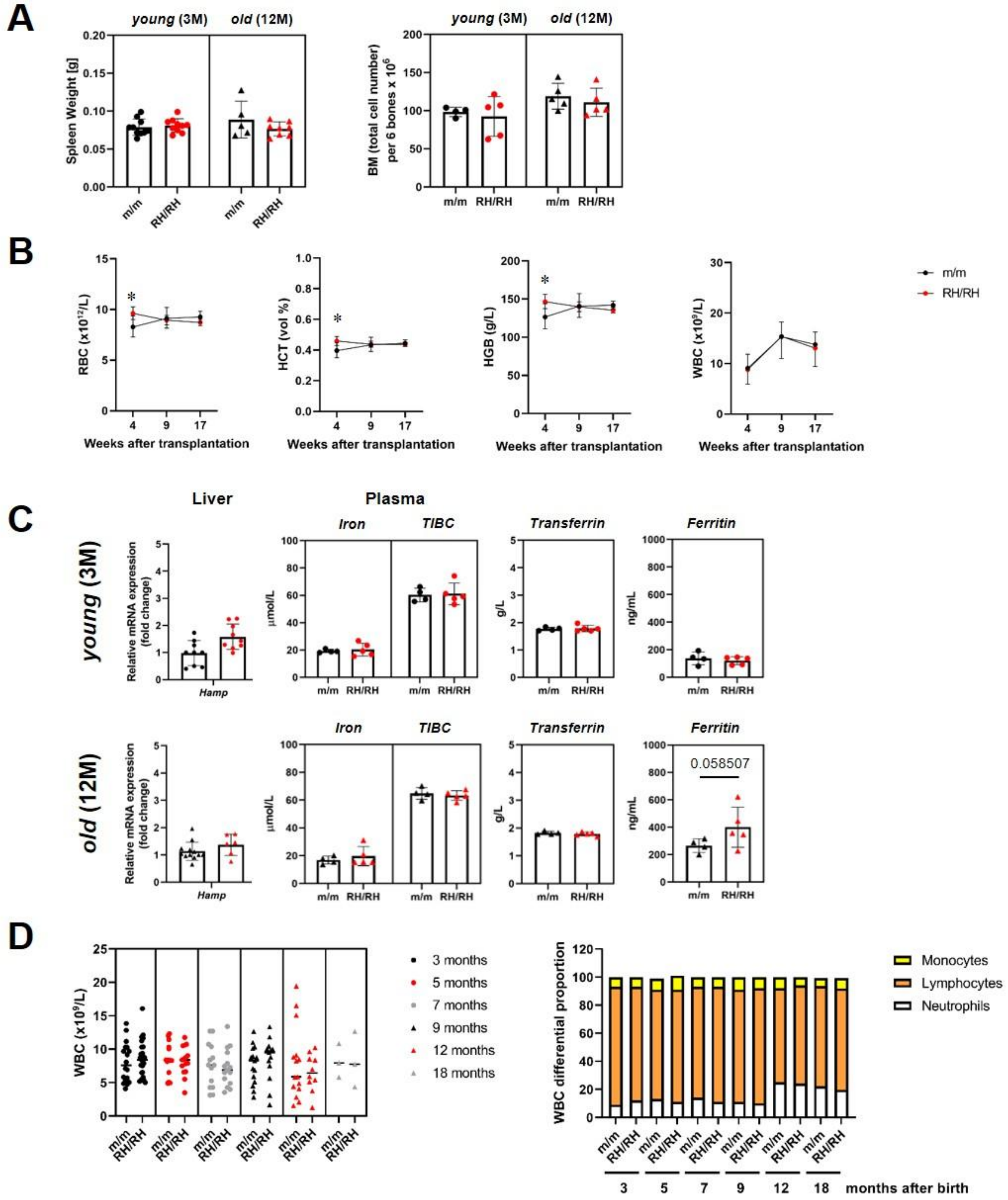

Figure S1. Phenotype of mice with the germline *Jak2*-R1063H mutation. (A) Spleen weight and absolute number of BM cells ($n \geq 4$ mice per group). (B) RBC, HCT, HGB and WBC counts after transplantation of unsorted BM cells to lethally irradiated recipients ($n \geq 10$ mice per group). (C) Hepatic hepcidin (*Hamp*) mRNA expression (normalized to *b-actin*) of young and old *Jak2*-R1063H mice (red) is presented as fold change relative to m/m (wt) control group ($n \geq 4$ mice per group). Iron parameters analyzed in plasma: total plasma iron (iron), total iron binding capacity (TIBC), transferrin

and ferritin ($n \geq 4$ mice per group). (D) White blood cells (WBC) counts and differential proportions in peripheral blood across aging in m/m (wt) and RH/RH mice. Analysis of peripheral blood was performed at 3, 5, 7, 9, 12 and 18 months of age ($n \geq 10$ per group, only for 18 months $n = 3$). Stacked bar graphs display the average proportion of monocytes (yellow), lymphocytes (orange), and neutrophils (white) within the total WBC population. No significant differences in WBC composition were observed between genotypes at any time point. Data are presented as mean ± SD and unpaired t-test with Welsch's correction was used for group comparison.

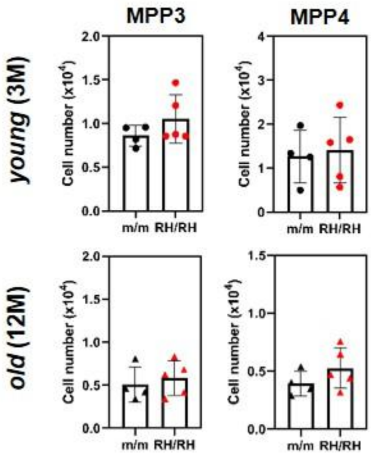

Figure S2. Quantification of absolute number of distinct stem and progenitor populations in BM. Lin^- $c\text{-}Kit^+$ $Sca\text{-}1^+$ $CD48^+$ $CD150^-$ $CD34^+$ $CD135^-$ (MPP3), Lin^- $c\text{-}Kit^+$ $Sca\text{-}1^+$ $CD48^+$ $CD150^-$ $CD34^+$ $CD135^+$ (MPP4) ($n \geq 4$ mice per group).

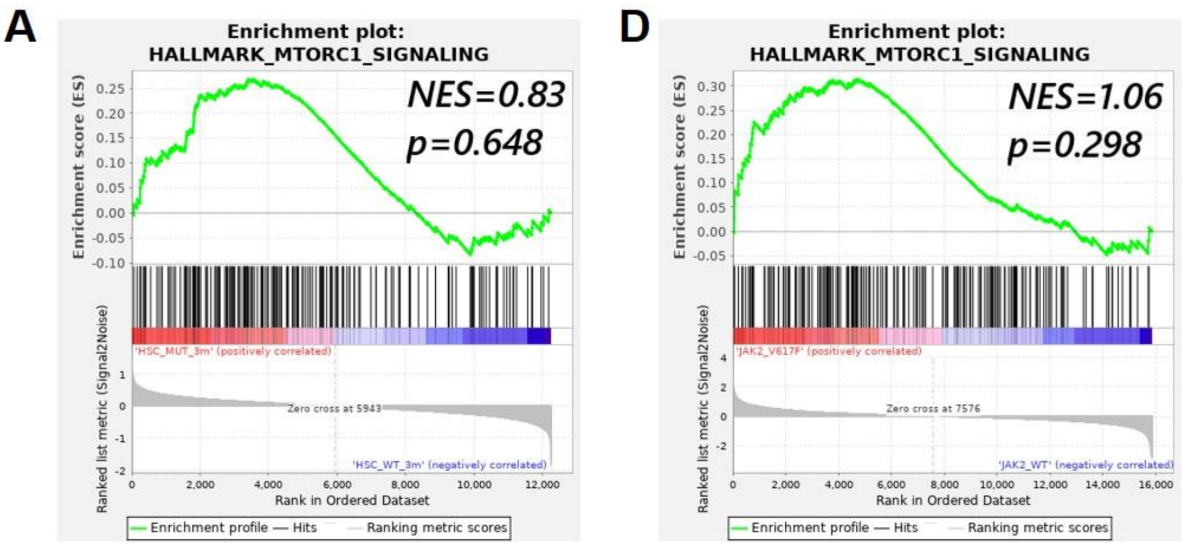

B

GOBP terms downregulated in V617F LSK population 8 weeks, analysis based on data from *Uras IZ et al, Blood* 2019 (project GSE123401)

young (8 weeks)		
Term name	Description	p-value
GO:0000278	Mitotic cell cycle	1.1E-4
GO:0007049	Cell cycle	2.3E-3
GO:0022402	Cell cycle process	1.7E-4
GO:0051301	Cell division	3.4E-4
GO:1903047	Mitotic cell cycle process	1.5E-3

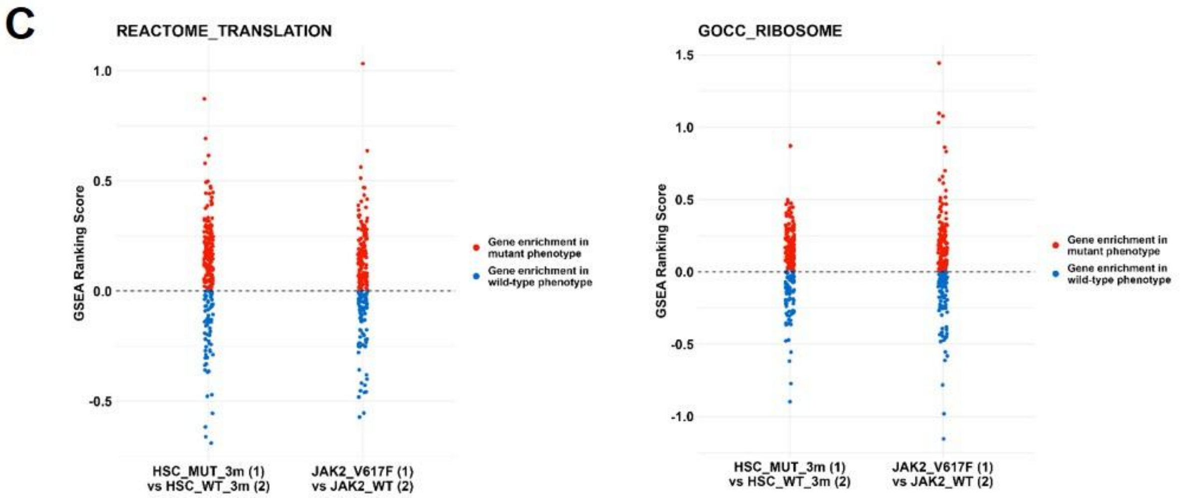

Figure S3. Expression analysis of *Jak2*-R1063H vs. wt HSCs and *JAK2*-V617F vs. wt LSK populations. (A) mTORC1 signaling pathway enriched (albeit insignificantly) in young (3M) RH/RH vs. m/m HSCs (from Hallmark gene sets by GSEA). (B to D) Data based on re-analysis of datasets from project GSE123401 (1); methodology is provided in **Supplemental Methods**. (B) GOBP terms downregulated in *Jak2*-V617F LSK vs. wt LSK population from 8-week-old mice. (C) Comparison of gene enrichment trends in young (3M) RH/RH vs. m/m HSCs and *Jak2*-V617F LSK vs. wt LSK populations from 8-week-old mice, in Reactome_Translation (M8229) and GOCC_Ribosome (M17089) GSEA pathways. In both cases, the majority of genes were enriched in mutant phenotype when compared to the wild-type. (D) mTORC1 signaling pathway enriched (albeit insignificantly) in young (8-wek-old) LSK of *Jak2*-V617F vs. wt (from Hallmark gene sets by GSEA).

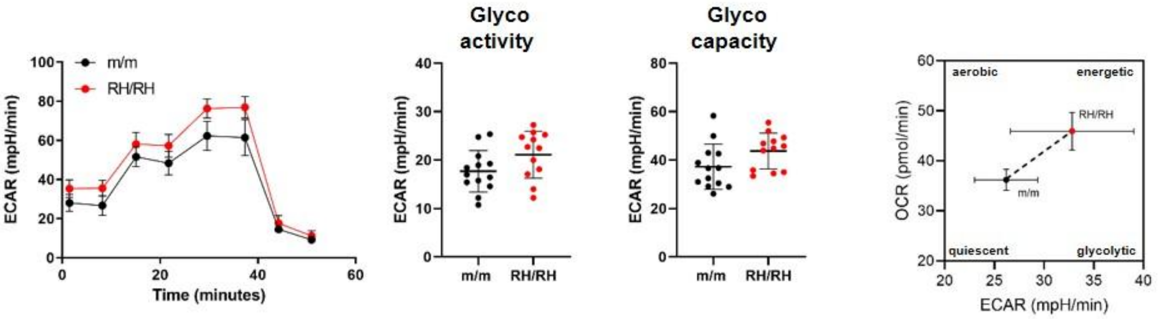

Figure S4. Seahorse analysis. Combined platelets ECAR profiles from m/m (wt) and RH/RH mice (young (3M)). Quantification of glycolytic activity and capacity is shown in the middle (*n* ≥ 6 mice per group). The higher metabolic activity in RH/RH platelets displayed as ECAR versus OCR (right).

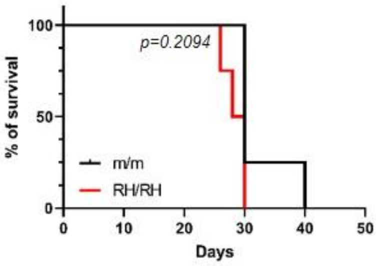

Figure S5. Kaplan-Meier survival analysis of m/m (*n* = 3) and RH/RH (*n* = 3) mice intravenously injected with 200 MLL-AF9 leukemic cells – a validation experiment. MLL-AF9 splenocytes isolated from a leukemic mouse were transplanted into non-irradiated 10- to 14-weeks old m/m (wt) and RH/RH recipients. Mantle-Cox test was used to assess statistical significance of obtained results (*p* = 0.2094).

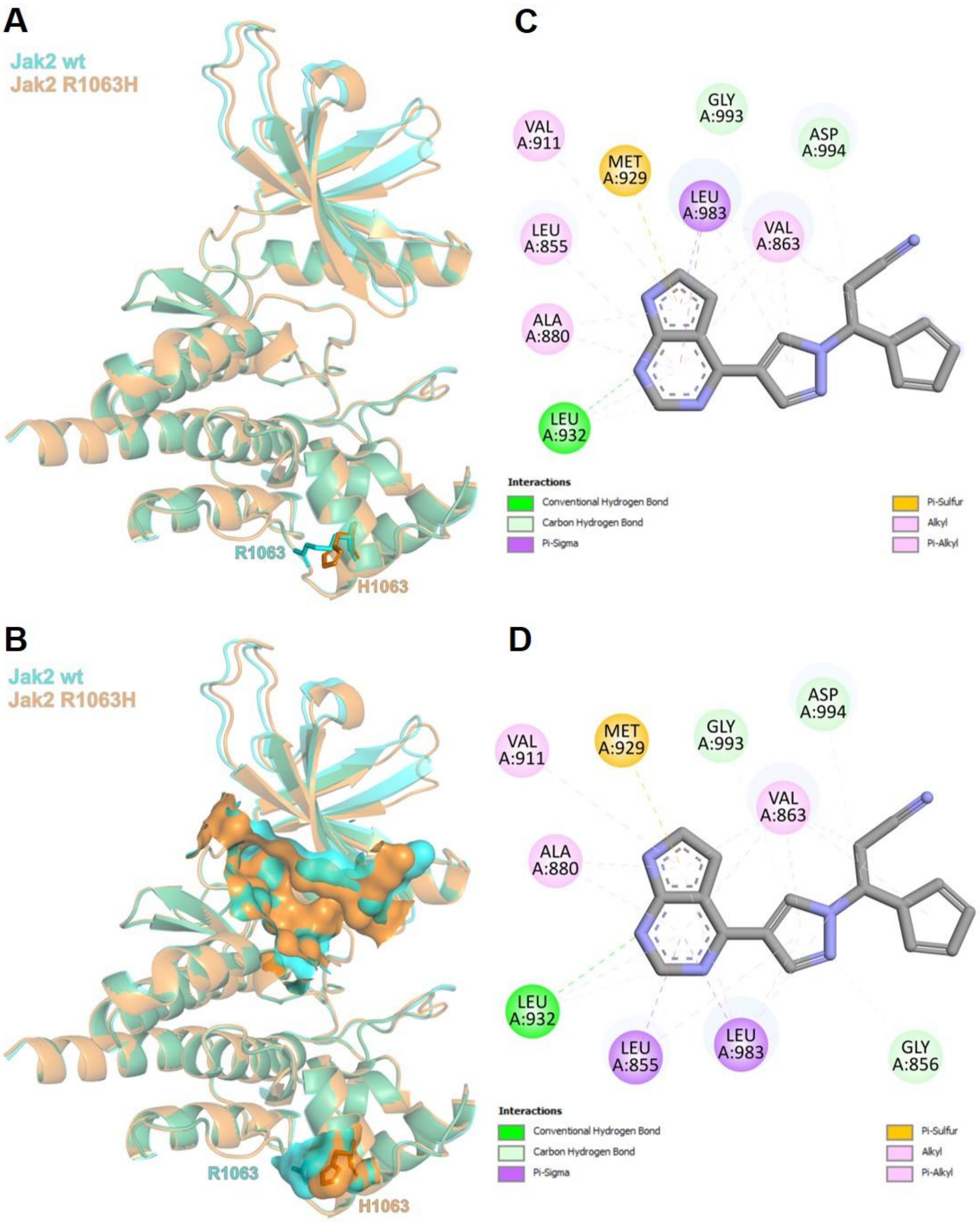

Figure S6. Modelling of the *JAK2*-R1063H mutation interactions with ruxolitinib. Modelling the R1063H mutation - located within the JH1 domain - revealed alterations in the active site geometry. Compared to the wt JAK2 (cyan) (A), in the R1063H mutant (orange), the shape and the size of the active cavity was remodeled, with narrower entrance to the cavity (B). The contracted state of the R1063H mutant was then compared to the JAK2-wt cocrystal with ruxolitinib (PDB: 6VGL), based on the homology alignment. Compared to the wt JAK2 (C), different interactions with the ruxolitinib in the R1063H mutant active site (D) were found (Leu855 shifted to stronger sigma interaction, additional carbon H-bond with Gly856), suggesting stronger binding into the mutant.

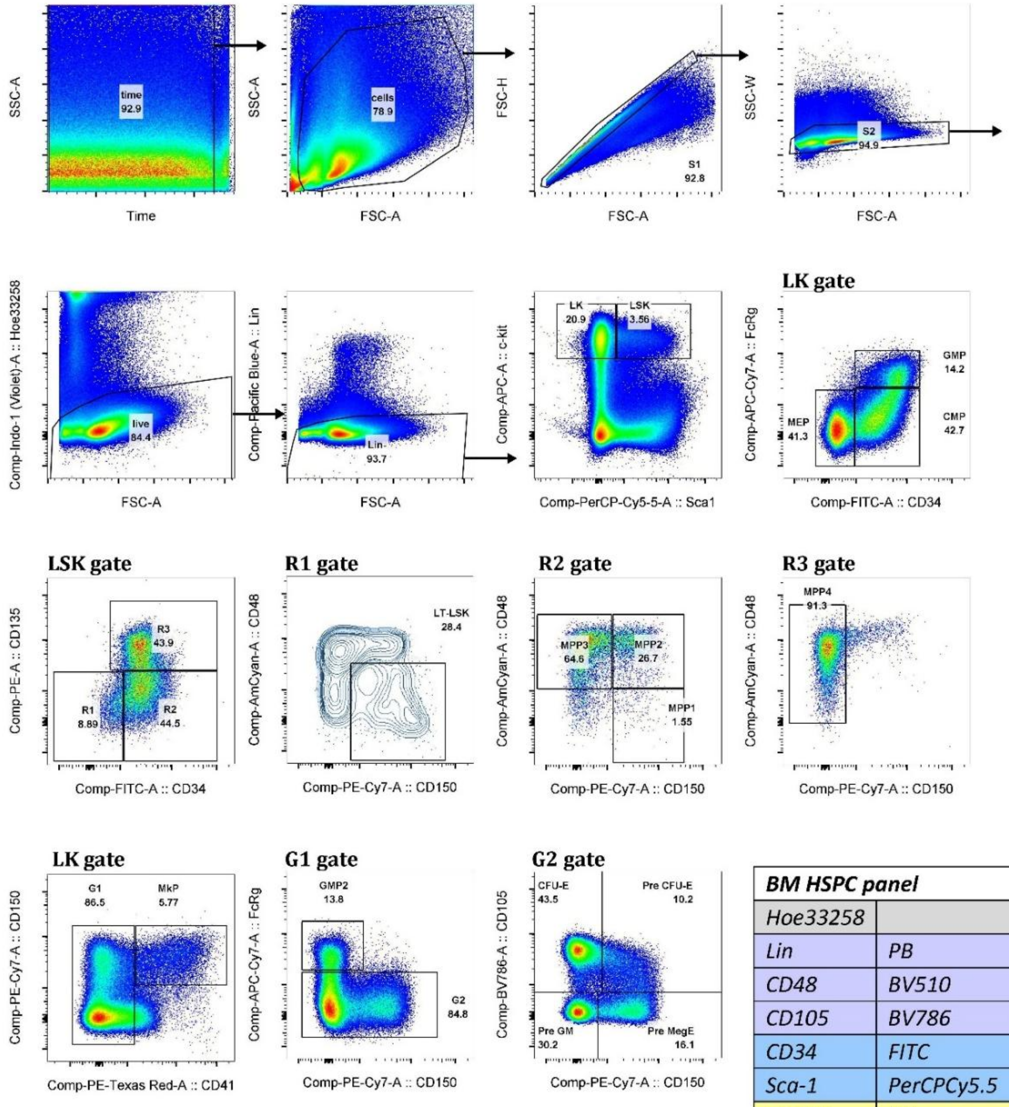

BM HSPC panel		*dilution*
Hoe33258		*10000x*
Lin	*PB*	*50x*
CD48	*BV510*	*200x*
CD105	*BV786*	*150x*
CD34	*FITC*	*150x*
Sca-1	*PerCPCy5.5*	*400x*
CD135	*PE*	*300x*
CD41	*PEDazzle594*	*150x*
CD150	*PC7*	*400x*
c-kit	*APC*	*800x*
FcRg	*APC-Cy7*	*300x*

Figure S7. Analysis of hematopoietic stem cells and myeloid progenitor cells. Gating strategy for flow cytometry analysis of BM subpopulations in young (3M) and old (12M) animals and details for used antibodies.

Reference

1. Uras IZ, Maurer B, Nivarthi H, Jodl P, Kollmann K, Prchal-Murphy M, et al. CDK6 coordinates JAK2 (V617F) mutant MPN via NF-κB and apoptotic networks. Blood. 2019;133(15):1677-90.
