# Supplementary material for: Germline Jak2-R1063H mutation interferes with normal hematopoietic development and increases risk of thrombosis and leukemic transformation
Source: Leukemia. 2025 Aug 21;39(11):2745–57. doi: 10.1038/s41375-025-02737-w (PMC12589134; doi:10.1038/s41375-025-02737-w)
Supplement: Supplementary file 3 — Supplemental Western Blots [file 41375_2025_2737_MOESM3_ESM.pdf]

Figure 1. B

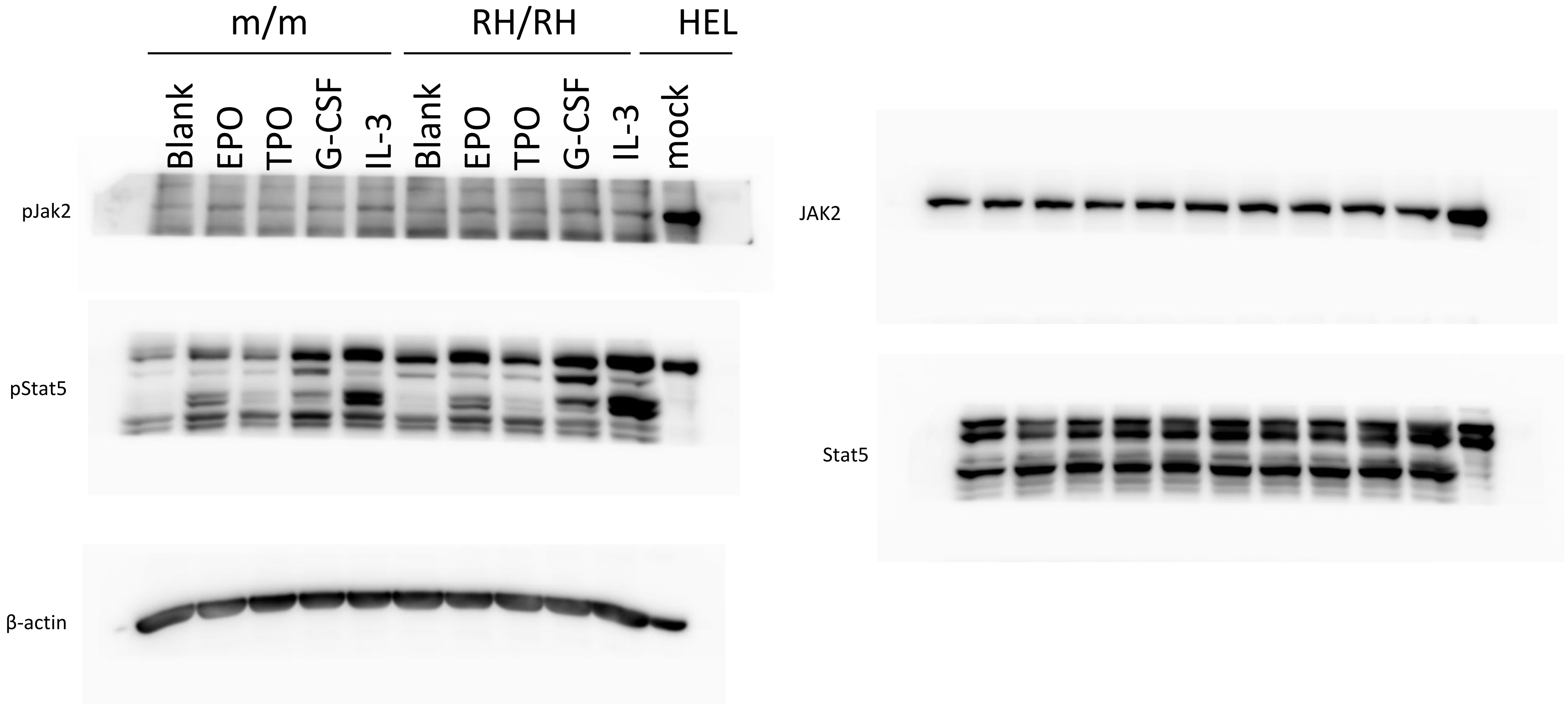

Increased baseline (blank line) and cytokine-stimulated Jak2/Stat5 activation in lysates of total BM cells obtained from RH/RH 3-months-old animals (n = 2, pooled cells), when compared to age-matched controls (n = 2), identified by Western blot analysis.

Figure 1. I (left)

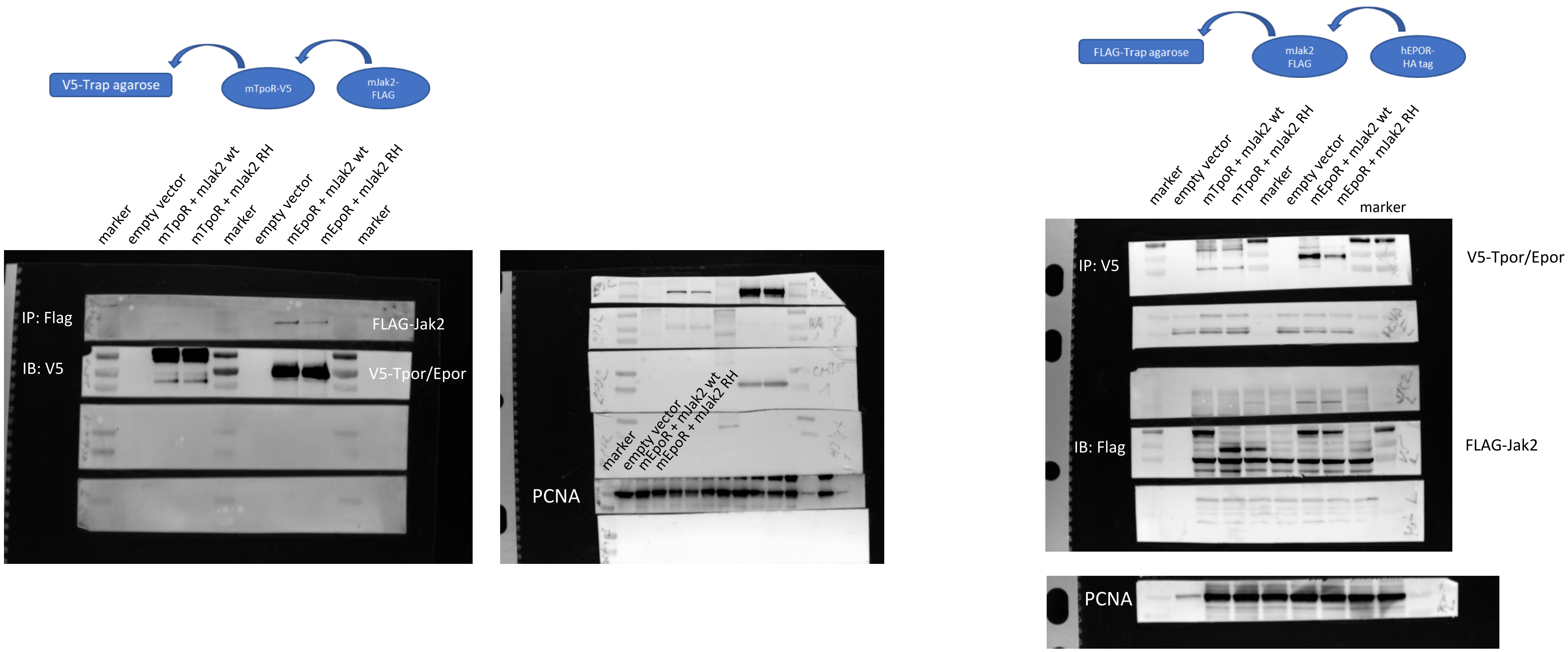

Altered coupling of mouse (m) Jak2 R1063H to hematopoietic receptors. Mouse Jak2-Flag mutants were transiently expressed in HEK293 cells in which mouse V5-tagged Epor/Tpor were stably expressed. Interaction was examined by co-immunoprecipitation with Flag/V5 antibody.

Figure 1. I (middle)

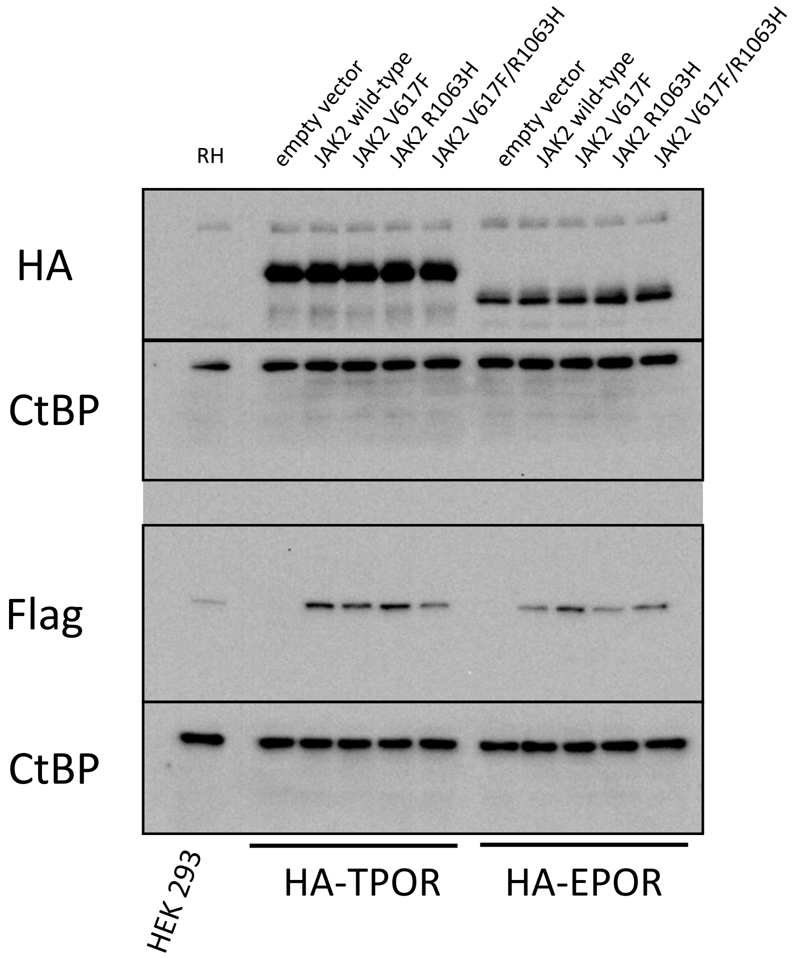

Altered coupling of human (h) JAK2 R1063H to hematopoietic receptors. Human JAK2-Flag mutants were transiently expressed in HEK293 cells in which human HA-tagged EPOR/TPOR were stably expressed. Interaction was examined by co-immunoprecipitation with Flag antibody.

Further information about vectors and experiment set-up can be also found:
Mambet C, Babosova O, Defour J-P, Leroy E, Necula L, Stanca O, et al. Cooccurring JAK2 V617F and R1063H mutations increase JAK2 signaling and neutrophilia in myeloproliferative neoplasms. *Blood*. 2018;132(25):2695-9.

Figure 3. C (young 3M)

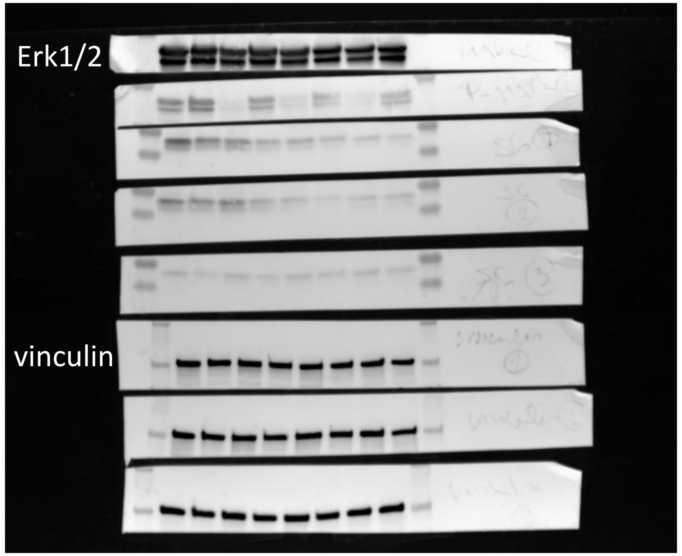

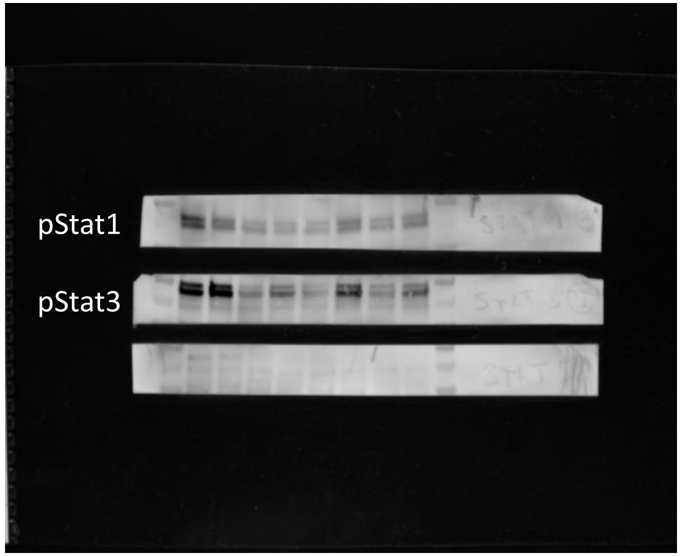

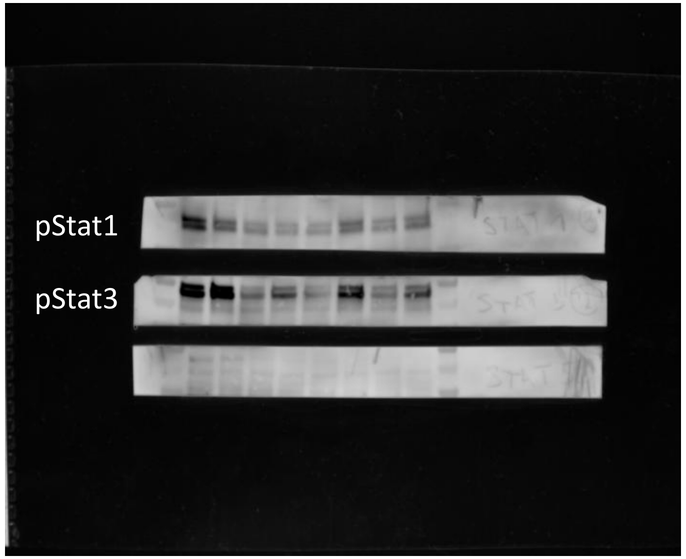

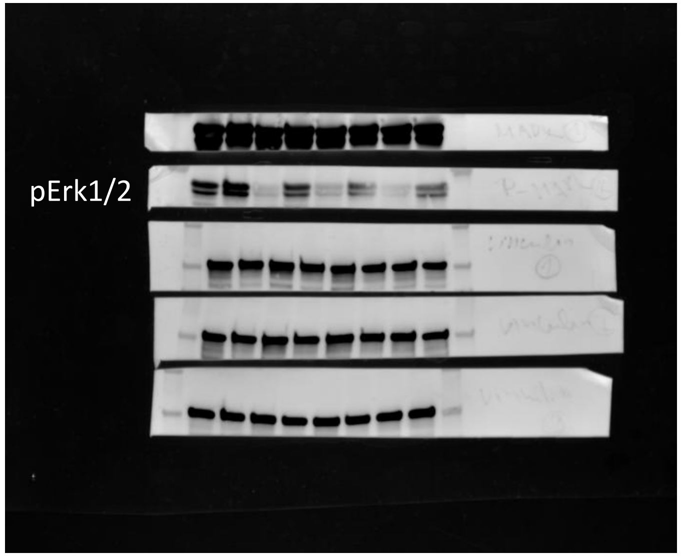

Phosphorylation of Stat1 (Tyr 694), Stat3 (Tyr 705) and Erk1/2 (Thr 202/Tyr 204) proteins in m/m (wt) and RH/RH c-Kit⁺ enriched fraction of total BM. Total Erk protein and Vinculin served as loading control. Western blot shows the baseline (blank line) and cytokine-stimulated protein activities in protein lysates from enriched c-kit⁺ cells, starved for 40 hours and unstimulated or stimulated with indicated cytokines for 15 minutes.

Figure 3. C (old 12M)

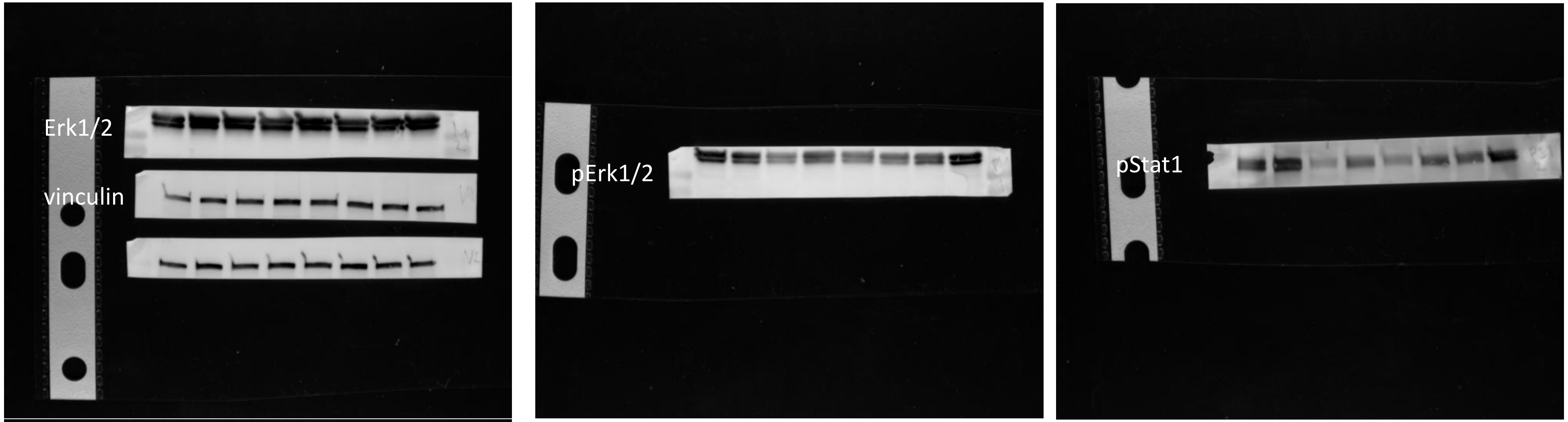

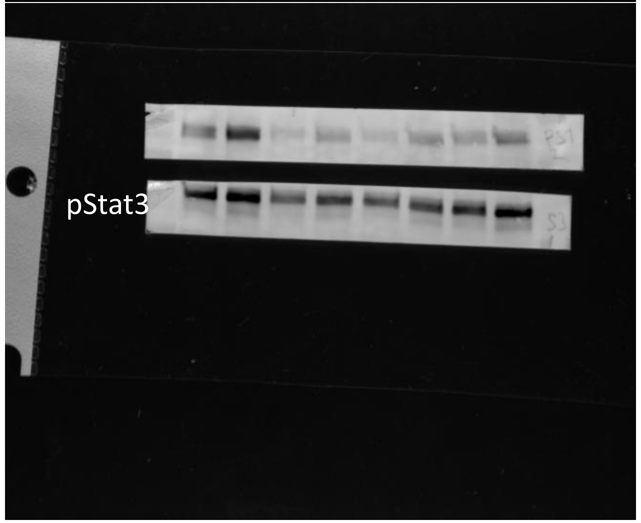

Phosphorylation of Stat1 (Tyr 694), Stat3 (Tyr 705) and Erk1/2 (Thr 202/Tyr 204) proteins in m/m (wt) and RH/RH c-Kit^{+} enriched fraction of total BM. Total Erk protein and Vinculin served as loading control. Western blot shows the baseline (blank line) and cytokine-stimulated protein activities in protein lysates from enriched c-kit^{+} cells, starved for 40 hours and unstimulated or stimulated with indicated cytokines for 15 minutes.
